# Integrated transcriptomic and metabolic analyses provide insights into the maintenance of embryogenic potential and the biosynthesis of phenolic acids and flavonoids involving transcription factors in *Larix kaempferi* (Lamb.) Carr.

**DOI:** 10.3389/fpls.2022.1056930

**Published:** 2022-11-17

**Authors:** Junchen Wang, Lifeng Zhang, Liwang Qi, Shougong Zhang

**Affiliations:** ^1^ State Key Laboratory of Tree Genetics and Breeding, Research Institute of Forestry, Chinese Academy of Forestry, Beijing, China; ^2^ Key Laboratory of Tree Breeding and Cultivation, National Forestry and Grassland Administration, Research Institute of Forestry, Chinese Academy of Forestry, Beijing, China

**Keywords:** embryogenic potential maintenance, embryogenic callus, non-embryogenic callus, transcriptomic, metabolomic, phenolic acids and flavonoids

## Abstract

Somatic embryogenesis (SE) techniques have been established for micropropagation or basic research related to plant development in many conifer species. The frequent occurrence of non-embryogenic callus (NEC) during SE has impose constraints on the application of somatic embryogenesis SE in *Larix kaempferi* (Lamb.) Carr, but the potential regulatory mechanisms are poorly understood. In this study, integrated transcriptomic and metabolomic analyses were performed in embryogenic callus (EC) and NEC originating from a single immature zygotic embryo to better decipher the key molecular and metabolic mechanisms required for embryogenic potential maintenance. The results showed that a total of 13,842 differentially expressed genes (DEGs) were found in EC and NEC, among which many were enriched in plant hormone signal transduction, starch and sucrose metabolism, phenylpropanoid biosynthesis, flavonoid biosynthesis, and the biosynthesis of amino acids pathways. Metabolite profiling showed that 441 differentially accumulated metabolites (DAMs) were identified in EC and NEC. Both EC and NEC had vigorous primary metabolic activities, while most secondary metabolites were upregulated in NEC. Many totipotency-related transcription factor (TF) genes such as *BBMs*, *WUSs*, and *LEC1* showed higher expression levels in EC compared with NEC, which may result in the higher accumulation of indole 3-acetic acid (IAA) in EC. NEC was characterized by upregulation of genes and metabolites associated with stress responses, such as DEGs involved in jasmonic acid (JA) and ethylene (ETH) biosynthesis and signal transduction pathways, and DEGs and DAMs related to phenylpropanoid and flavonoid biosynthesis. We predicted and analyzed TFs that could target several key co-expressed structural DEGs including two *C4H* genes, two *CcoAOMT* genes and three *HCT* genes involved in phenylpropanoid and flavonoid biosynthesis. Based on the targeted relationship and the co-expression network, two ERFs (Lk23436 and Lk458687), one MYB (Lk34626) and one C2C2-dof (Lk37167) may play an important role in regulating phenolic acid and flavonoid biosynthesis by transcriptionally regulating the expression of these structural genes. This study shows an approach involving integrated transcriptomic and metabolic analyses to obtain insights into molecular events underlying embryogenic potential maintenance and the biosynthesis mechanisms of key metabolites involving TF regulation, which provides valuable information for the improvement of SE efficiency in *L. kaempferi*.

## Introduction

*Larix kaempferi* (Lamb.) Carr is one of the most important fast-growing plantation species in China. It is widely distributed in alpine coniferous forests in northeastern and northern China, Inner Mongolia, and southwestern China (Zhang et al., 2010; Fan et al., 2020; Wu et al., 2021) and plays a substantial role in forest timber production as well as in ecological construction (Fan et al., 2020). However, *L. kaempferi* features a long-life span and complex genetic background, making it difficult to breed new lines with conventional methods especially by seed propagation (Zhang et al., 2014a; Zhang et al., 2021a). Therefore, to establish and develop efficient reproduction technologies is of great significance for multiplying true-to-type selected varieties with desired characteristics. Conifer somatic embryogenesis (SE) technology is powerful in the mass vegetative propagation of elite varieties or genotypes with desired traits, which has been applied in *Larix* species for more than three decades since it was first reported in *Picea abies* (Hakman et al., 1985) and *L. decidua* (Nagmani and Bonga, 1985). To date, SE technology has been regarded as an ideal system for basic research about gymnosperm growth and development and the related regulatory mechanisms (Zhang et al., 2021a). For example, stable gene transformation technology has been established in *L. kaempferi* SE system and has been utilized to have multiple genes functionally validated (Fan et al., 2020; Fan et al., 2021; Kang et al., 2021). However, micropropagation through SE technology has not been widely applied to plant regeneration in *L. kaempferi* due to several problems such as difficult embryonal-suspensor mass (ESM) induction, frequent abnormal embryo development, and challenges *in vitro* synchronization (Zhang et al., 2012b; Zhang et al., 2021a). Therefore, further knowledge is required to address these problems and realize the full potential of SE.

SE is a complex process of embryo development involving cell dedifferentiation and reprogramming to regenerate whole plants, which is regulated by a network of hundreds of genes (Mordhorst et al., 1997; Wang et al., 2020). Many studies focusing on plant SE were conducted in some short-lived angiosperms such as *Cucumis sativus* (Xue et al., 2022), *Gossypium hirsutum (* Guo et al., 2019 *;* Kumar et al., 2021 *)*, and *Fragaria vesca* (Liu et al., 2022), and most in-depth knowledge of SE can be only obtained from Arabidopsis (Horstman et al., 2017; Karami et al., 2021; Li et al., 2022b; Paul et al., 2022). However, little is known from perennials including gymnosperm. Despite the promising achievements obtained in *Picea abies* and *P. glauca* (Klimaszewska et al., 2011; Varis et al., 2018; Nakamura et al., 2020), in most conifers including *L. kaempferi*, the whole process of SE can only be initiated from immature seeds, in practice immature zygotic embryos (Ávila et al., 2022). In the ESM induction of *L. kaempferi*, a cytological characteristic is that two distinct types of callus may be generated: embryogenic callus (EC) and non-embryogenic callus (NEC), during which the cells must dedifferentiate, activate their cell division cycle, and reorganize their physiological and metabolic states (Kumaravel et al., 2017; Wang et al., 2020). EC and NEC are different in their appearance and biochemical properties, but both of them can be maintained in medium with 2,4-Dichlorophenoxyacetic acid (2,4-D) and 6-Benzylaminopurine (6-BA). NEC are characterized by unorganized, dedifferentiated, continuously dividing cell masses (Kumar et al., 2021), whereas EC cells are in a state of vigorous proliferation and differentiation. These results furtherly reflect the distinctions in embryogenic potentials between EC and NEC. For *L. kaempferi*, EC consists of proembryogenic masses and have the ability to develop into rapidly proliferating early somatic embryos and eventually form a whole plant in growth medium, namely embryogenic potential (Bonga et al., 2009; Elhiti et al., 2013), while NEC have no embryogenic development ability. Nevertheless, limited knowledge is known about the molecular mechanisms underlying the formation of EC and NEC as well as the distinct embryogenic potentials between EC and NEC, which may be crucial for increasing the initiation rate of SE.

Besides the unexpected occurrence of NEC during ESM induction, EC may be frequently transformed to NEC during sub-culture process, which combinedly impose constraints on the stability of SE system in *L. kaempferi* (Zhang et al., 2010). Therefore, the maintenance of embryogenic state is crucial for the proper embryo development and global SE efficiency. However, the potential regulatory networks involved in these processes are poorly understood. In angiosperms, genes including *SOMATIC EMBRYOGENESIS RECEPTOR KINASE* (*SERK*) family members, *BABY BOOM* (*BBM*), *WUSCHEL* (*WUS*), and *LEAFY COTYLEDON (LEC)* are involved in the transition from NEC to EC (Gruel et al., 2018; Mendez-Hernandez et al., 2019) and the overexpression of these genes can increase embryo formation frequency (Lotan et al., 1998; Boutilier et al., 2002; Zuo et al., 2002; Lowe et al., 2016; Horstman et al., 2017; Li et al., 2022b), but the degree of the increase differed between monocot and dicot systems (Srinivasan et al., 2007; Zhang et al., 2014b; Florez et al., 2015; Kumar et al., 2021). Moreover, it has been reported that genes related to stress response such as *abscisic aldehyde synthesis enzyme 2* (*ABA2*), *abscisic acid insensitive 3* (*ABI3*), *jasmonate ZIM-domain 1* (*JAZ1*), *late embryogenesis abundant protein 1* (*LEA1*), and transcription factors like AUX/IAAs, NACs, WRKYs, MYBs, and ERFs were also involved in callus induction (Jin et al., 2014; Fehér, 2015; Guo et al., 2019; Salaün et al., 2021). In conifers, some studies have compared EC and NEC of several species including *L. kaempferi* (Zhang et al., 2010), *P. balfouriana* (Li et al., 2014), *Pinus radiata* (Bravo et al., 2017), *Pseudotsuga menziesii* (Gautier et al., 2019), *P. abies* (Nakamura et al., 2020), and *Pinus pinaster* (Ávila et al., 2022) from the perspectives of miRNA and their targeted gene expression, DNA methylation, histone modification, cytological or biochemical characteristics, and transcriptomic or proteomic profiling. Although a few putative gene markers for embryogenic cells such as *BBM* and *WUS* as mentioned above have been highlighted in these studies, whether these markers are universal for other plant species remains empirical.

The application of transcriptomics in diverse tree species have been very successful in providing a detailed gene expression profile of plant cells, which is important in understanding basic functions in tree biology (Ávila et al., 2022). In *L. kaempferi*, the availability of genetic and genomic resources has largely allowed its use in functional genomics approaches (Sun et al., 2022). Similar to transcriptomics or proteomics, metabolomics is an emerging omics technology aimed at identifying and quantifying endogenous molecule metabolites, the final products of cell biological regulation process (Lin et al., 2020). In recent years, “multi-omics” strategies have provided new insights in revealing underlying mechanisms in plant growth and development as well as in plant responses to external stresses (Xiao et al., 2021; Zhang et al., 2021c; Li et al., 2022a; Wang et al., 2022b). The combined analysis of transcriptome and metabolome data can be used to predict the gene function involved in the targeted metabolic pathway and simultaneously provide supporting information for gene mining (Fukushima et al., 2009). In conifers, the integrated analysis of the two omics were used in several species such as *Pinus pinaster* (Cañas et al., 2015), *Pinus radiata* (Escandón et al., 2017), *P. abies* (Dobrowolska et al., 2017; Blokhina et al., 2019), *Pinus taeda* (Mao et al., 2021), *Pinus massoniana* (Cai et al., 2021), and *L. olgensis* (Zhang et al., 2021b) to address the regulatory mechanisms underlying some problems in plant systems biology including tree responses to environmental changes or disease, the biosynthesis of targeted metabolites, and somatic embryo germination. Given the fact that SE induction is an initiation phase regulated by a complex network of numerous genes (Mordhorst et al., 1997; Wang et al., 2020), there must also be many metabolic activities functioning in this process, which can be reflected by the differences between EC and NEC. Moreover, the integration of transcriptomics and metabolomics may offer notable advantages to identify the biosynthetic mechanisms of key metabolic pathways underlying the formation of EC and NEC. However, no previous studies have compared EC and NEC using integrated transcriptomic and metabolic analysis.

The morphology of EC and NEC of *L. kaempferi* was addressed elsewhere (Zhang et al., 2010). In this work, transcriptomics and metabolomics were carried out on EC and NEC produced in the process of transdifferentiation. The aim of this study was to explore the gene expression and metabolic differences between EC and NEC and furtherly reveal the molecular mechanisms underlying key metabolites accumulation as well as embryogenic potential maintenance. To the best of our knowledge, this work is the first study in conifers trying to reveal the molecular basis of small-molecule metabolite biosynthesis during SE induction. Our results will provide new insights into conifer SE, with potential value to increase SE efficiency in *L. kaempferi* breeding program.

## Materials and methods

### Plant material

**Figure 1:**
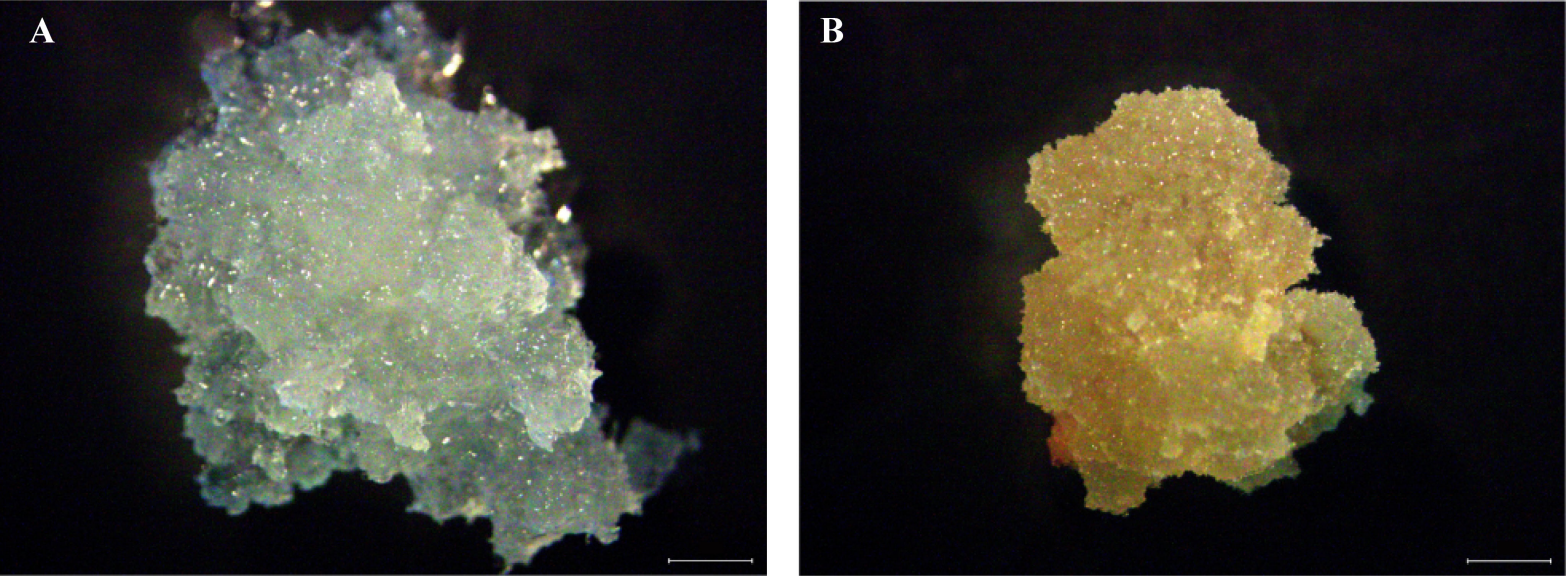
Embryogenic callus (EC) and non-embryogenic callus (NEC) in *L. kaempferi*. **(A)** EC. **(B)** NEC. Scale bar: 1mm.

The embryonal-suspensor mass (ESM) was induced from *L. kaempferi* immature zygotic embryos on solid induction medium containing 450 mg/L glutamine, 500 mg/L casein hydrolysate, 1,000 mg/L inositol, 30 g/L sucrose, 3 g/L Gelrite, 10 µM 2,4-D, and 3.6 µM 6-BA in darkness at approximatively 23 °C (Zhang et al., 2014a; Zhang et al., 2021a). Two types of callus, EC and NEC, were found to emerge from a single immature zygotic embryo during this process (named cell line C6). Then, EC and NEC were sub-cultured every three weeks on solid proliferation medium supplemented with 450 mg/L glutamine, 500 mg/L casein hydrolysate, 1000 mg/L inositol, 30 g/L sucrose, 3 g/L Gelrite, 0.5 µM 2,4-D, and 0.18 µM 6-BA (Zhang et al., 2014a; Zhang et al., 2021a). After being sub-cultured for 15 days, the proliferating material of EC and NEC was collected (**Figure 1**), frozen in liquid nitrogen, and stored at -80 °C until use.

### RNA sequencing and functional annotation

RNA was extracted from three biological replicates of both EC and NEC. Samples were ground to fine powder. The total RNA was isolated using the RNAiso Plus and RNAiso-mate for Plant Tissue kits (Takara, Japan) according to the manufacturer’s instructions and was treated with DNase (Takara, Japan) to remove DNA. To ensure the accuracy of the sequencing data, the total RNA samples were prepared as follows. RNA degradation and contamination was monitored on 1% agarose gels and RNA purity was checked using the NanoPhotometer^®^ spectrophotometer (IMPLEN, CA, USA). RNA concentration was quantified using Qubit^®^ RNA Assay Kit in Qubit^®^2.0 Flurometer (Life Technologies, CA, USA). Finally, RNA integrity was assessed using the RNA Nano 6000 Assay Kit of the Bioanalyzer 2100 system (Agilent Technologies, CA, USA).

The cDNA library for each sample was sequenced on the Illumina sequencing platform by Metware Biotechnology Co., Ltd. (Wuhan, China). After that, many high-quality raw reads were selected, and clean reads were obtained by removing low-quality ones. All the clean reads were mapped separately to the *L. kaempferi* V1.0 genome using HISAT2 software. The basic local alignment search tool (BLAST) was used to annotate the functions of unigenes against protein databases, including Non-redundant Protein Database (NR), Gene ontology (GO), Kyoto Encyclopedia of Genes and Genomes (KEGG), TrEMBL, Kyoto Encyclopedia of Genes and Genomes (KOG), Clusters of Orthologous Groups (COG), Protein Family Database (Pfam), and Non-redundant Protein Sequence Database (SwissProt) using BLAST with an e-value threshold of 1×10^−5^.

### Identification of differentially expressed genes

To verify the transcription expression levels of all samples, fragments per kilobase of transcript per million mapped reads (FPKM) were calculated by featureCounts v1.6.2 to quantify the expression level of gene. Subsequently, differentially expressed genes (DEGs) between the two groups were filtered by DESeq2 software with |log2 fold change (FC)| ≥ 1 and false discovery rate (FDR) <0.01. The gene expression patterns were obtained through hierarchical clustering analysis. The GO and KEGG pathway enrichment analysis of DEGs was performed based on the hypergeometric test.

### Quantitative real-time polymerase chain reaction (qRT-PCR) validation of RNA-seq data

The accuracy of RNA-seq data was verified using qRT-PCR. Total RNA was extracted from the calluses with RNAiso Plus and RNAiso-mate for Plant Tissue kits (TaKaRa, Dalian, China), after which 1 µg RNA was reverse transcribed using the PrimeScript™ RT Reagent Kit with gDNA Eraser (Perfect Real Time) (TaKaRa). The expression levels of selected genes were analyzed in a qRT-PCR assay conducted using a SYBR Premix Ex Taq Kit (TaKaRa) and the CFX96™ Real-Time

System. All primer sequences for these genes were shown in **Supplementary Table 1**. The specificity of the qRT-PCR primers was confirmed by separating the products on agarose gels and by sequencing (**Supplementary Figure 1**). The relative gene expression in each sample was calculated by the 2^−△△ct^ method against *LkEF1A1* (GenBank accession: JR153706) (Zhang et al., 2012a). SPSS 26.0 was used for ANOVA to test the significant differences of relative gene expression levels between EC and NEC and *P* < 0.05 was the threshold for significance.

### Metabolomic analysis

Metabolomic analysis was conducted for three biological replicates of both EC and NEC. The freeze-dried calluses of *L. kaempferi* were homogenized in a ball mill. 100 mg powder was weighed and dissolved with 1.2 ml 70% methanol solution, vortexed 30 seconds every 30 minutes for 6 times in total, and placed in a refrigerator at 4°C overnight. After centrifugation at 12000 rpm for 10 min, the extracts were filtrated (0.22 μm pore size) for following ultra-performance liquid chromatography-tandem mass spectrometry (UPLC-MS/MS) analysis by Metware Biotechnology Co., Ltd. (Wuhan, China).

Multiple reaction monitoring (MRM) was performed as previously described (Mao et al., 2021). Multivariate principal component analysis (PCA) and orthogonal partial least squares-discriminant analysis (OPLS-DA) were conducted using the base package and “MetaboAnalystR” in R. The multivariate analysis of variable importance in projection (VIP) in the OPLS-DA model was used to initially screen differentially accumulated metabolites (DAMs). The DAMs were identified based on VIP ≥ 1 and fabsolute log2FC (fold change) ≥ 1. K-means analysis and heatmap analysis based on hierarchical clustering were performed in R. Functional annotation and enrichment analysis of the DAMs were conducted based on the KEGG database (Du et al., 2021).

### Correlation analysis of the transcriptome and metabolome data

Based on the transcriptome and metabolome data, Pearson’s correlation analysis was performed to explore the correlations between the DEGs and DAMs. The DEGs and DAMs were mapped to the KEGG pathway database to obtain their common pathway information. Only the detected correlations with a Pearson correlation coefficient (PCC)> 0.9 and *P*-value < 0.01 were selected. Subsequently, the related networks were visualized using the Cytoscape software.

### Prediction of key transcription factors (TFs)

The DEGs highly related to key DAMs synthesis between EC and NEC were selected as target genes in this analysis. We used TBtools (Chen et al., 2020) to extract 2,000-bp sequences before the CDSs of the key genes as the potential promoter sequences based on the *L. kaempferi* genome data (Sun et al., 2022). The promoter sequences of these genes were uploaded to the online database PlantTFDB (http://planttfdb.gao-lab.org/) (Tian et al., 2020) and the potential TFs regulating target genes were predicted based on the Arabidopsis genome. BLAST program was employed to identify the best match between Arabidopsis and *L. kaempferi* genome. The significantly differentially expressed TFs between EC and NEC were selected as candidate TFs regulating the synthesis of key DAMs.

## Results

### Metabolomic profiling analysis

**Figure 2:**
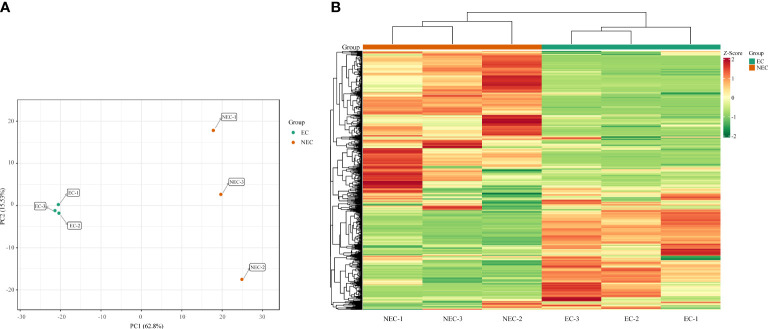
Principal component analysis (PCA) score plots and heat map of metabolite profile. **(A)** PCA score plots with each point representing an independent biological replicate; **(B)** Heatmap of metabolite abundance of EC and NEC according to hierarchical cluster analysis, where green, yellow, and red indicate low, intermediate, and high accumulation, respectively.

A widely targeted metabolomic analysis was performed to produce a metabolic profile by using UPLC-MS/MS system. An amount of 835 metabolites were identified and were divided into 14 categories, mainly containing lipids, flavonoids, phenolic acids, and amino acids and derivatives (**Supplementary Tables 2**, **3**). Overall, primary metabolites showed a similar amount with secondary metabolites in all the detective metabolites, indicating that *L. kaempferi* callus possessed both vigorous primary and secondary metabolic activities. We used principal component analysis (PCA) to reveal the metabolite differences between EC and NEC. The results showed that PC1 and PC2 cumulatively explained 78.33% of the total variance of the samples (62.80% and 15.53% for PC1 and PC2, respectively), and all samples were grouped into two distinct clusters (**Figure 2A**). Moreover, the hierarchical clustering results also suggested the high repeatability of the biological replicates in each group and the significant differences in metabolites between EC and NEC (**Figure 2B**).

### DAMs identification and enrichment analysis

The results of Orthogonal partial least squares-discriminant analysis (OPLS-DA) analysis and response permutation testing (RPT) were shown in **Supplementary Figures 2A, B**. DAMs between EC and NEC were screened and identified by using univariate and multivariate statistical analysis with threshold values of VIP ≥ 1 and fold change ≥ 2 or ≤ 0.5. Overall, 441 DAMs were obtained between the two groups, among which 177 metabolites were downregulated while 264 were upregulated in NEC compared to EC (**Supplementary Figure 2C**; **Supplementary Table 4**). Moreover, 30 and 79 metabolites were specifically accumulated in EC and NEC, respectively (**Supplementary Table 4**). The results of top ten metabolites with the largest fold change were shown in **Supplementary Figure 2D**, in which gallocatechin*, lysoPC 18:1*, p-coumaroylquinic acid-4’-O-glucuronide, nortrachelogenin 4-O-β-D-glucoside, Procyanidin C2*, catechin-catechin-catechin, and wogonin were the most differentially accumulated compounds between EC and NEC.

The DAMs obtained in this study contained a larger proportion of secondary metabolites than primary metabolites (**Table 1**). For primary metabolites including amino acids and derivatives, nucleotides and derivatives, lipids, and organic acids which accounted for relatively large proportions of all DAMs (175, 39.68%), 99 DAMs were downregulated while 76 were upregulated in NEC. For secondary metabolites including flavonoids, phenolic acids, lignans and coumarins, and alkaloids, 137 DAMs were upregulated while 63 were downregulated in NEC. There were 90 flavonoids and 82 phenolic acids differentially accumulated between EC and NEC, accounting for 20.41% and 18.59% of all the DAMs, respectively (39% in total). The 90 flavonoids were composed of 2 chalcones, 14 flavanols, 12 flavanones, 6 flavanonols, 24 flavones, 1 flavonoid carbonoside, and 31 flavonols, in which 70 DAMs were upregulated while 20 were downregulated in NEC. For phenolic acids, 55 DAMs were upregulated while 27 were downregulated in NEC (**Supplementary Table 4**).

**Table 1:** Categories of the 441 DAMs between EC and NEC of *L. kaempferi*.

Order	Category	Number of metabolites
1	Flavonoids	90
2	Phenolic acids	82
3	Amino acids and derivatives	59
4	Lipids	48
5	Organic acids	40
6	Nucleotides and derivatives	28
7	Lignans and Coumarins	25
8	Alkaloids	21
9	Tannins	11
10	Others	11
11	Saccharides and Alcohols	10
12	Vitamin	9
13	Terpenoids	7
	Total	441

The results of KEGG enrichment of the DAMs showed that DAMs were mainly mapped onto “flavonoids biosynthesis”, “flavone and flavonol biosynthesis”, “arginine and proline metabolism”, “phenylalanine metabolism” and “phenylpropanoid biosynthesis” (**Supplementary Figure 3**).

### Overview of RNA-seq and functional annotation

Six cDNA libraries from the EC and NEC of *L. kaempferi* were constructed and sequenced. A total of 297,123,278 raw reads and 276,606,454 clean reads were obtained. The average Q20 and Q30 values were 97.62 and 93.27%, respectively. The average of GC content was 44.76% (**Table 2**). Additionally, the overall mapped ratio exceeded 90% and the unique mapped ratio ranged from 90.36 to 91.88%. The overall expression patterns of all the samples demonstrated that the distribution of gene expression in all samples was similar, without extremely high/low expression in any sample (**Supplementary Figure 4A**). Moreover, the correlation between the samples showed the high repeatability of the three replicates within groups and the significant differences between EC and NEC (**Supplementary Figure 4B**). In general, the transcriptome sequencing quality and depth of the samples could well meet the necessary standard for the following analysis.

**Table 2:** Overview of transcriptome sequencing of complementary DNA from *L. kaempferi* callus.

Sample	Raw Reads	Clean Reads	Clean Base(G)	Q20 (%)	Q30 (%)	GC Content (%)	Mapped ratio (%)	Unique mapped ratio (%)
EC-1	50,451,060	47,142,820	7.07	98.11	94.14	44.09	91.87	86.69
EC-2	47,182,768	43,338,370	6.50	97.78	93.61	44.31	91.43	86.18
EC-3	50,148,592	47,178,166	7.08	97.6	93.12	44.10	91.88	86.81
NEC-1	46,096,912	43,091,380	6.46	97.53	93.12	45.05	90.79	85.05
NEC-2	55,128,588	50,941,694	7.64	97.14	92.37	45.49	90.36	83.82
NEC-3	48,115,358	44,914,024	6.74	97.57	93.25	45.49	90.70	84.26
Summary	29,7123,278	276,606,454	41.49	97.62	93.27	44.76	91.17	85.47

### DEGs identification and enrichment analysis

**Figure 3:**
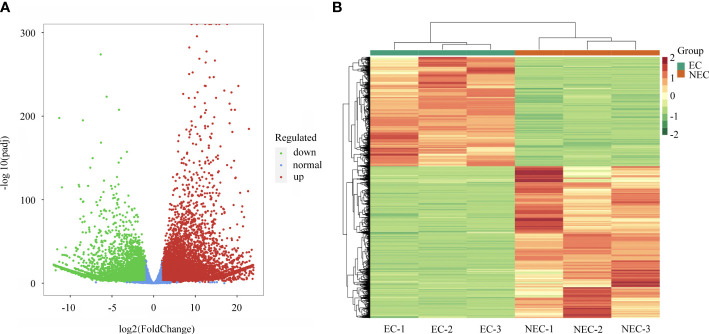
Analysis of differentially expressed genes (DEGs) between EC and NEC. **(A)** Volcano plot of DEGs between EC and NEC libraries, red and green dots represent the significantly upregulated and downregulated genes, respectively. **(B)** Heatmap of DEGs between EC and NEC libraries based on hierarchical clustering analysis.

The FPKM method was applied to calculate the gene expression and DEGs were screened with adjusted *P* values < 0.05. A total of 13,842 significant DEGs were identified between EC and NEC (**Supplementary Table 5**). Among them, 5,798 genes were downregulated while 8,044 genes were upregulated in NEC compared to EC (**Figure 3A**). The expression patterns of EC and NEC libraries were compared, revealing that the colors of EC-1, EC-2 and EC-3 were similar and that these libraries were classified into the same cluster (**Figure 3B**).

To better understand the biological functions of DEGs, we functionally categorized the DEGs using GO and KEGG enrichment analysis. For GO analysis, the subcategory with the highest enrichment degree was “cellular anatomical entity” (7,576 genes), followed by “cellular process” (5,780 genes). “Cellular process” and “metabolic process” (4,791 genes) were the two subcategories with the highest enrichment degree in the biological process category. In the molecular function category, the “binding” (5,611 genes) and “catalytic activity” (5,468 genes) subcategories represented the two largest groups with the highest enrichment degrees (**Supplementary Figure 5A**). Similar to the functional categories, three GO-directed acyclic graphs were constructed. The number of significantly enriched GO terms obtained in the graphs of the biological process, cellular component, and molecular function category were 5, 8, and 6, respectively (**Supplementary Figure 5B**).

For KEGG analysis, the significant DEGs between EC and NEC libraries were assigned into five different KEGG terms in the order from top to down: metabolism, genetic information processing, environmental information processing, cellular processes, and organismal systems. A total of 4,598 DEGs were assigned into 134 KEGG pathways, in which the most DEGs were involved in “metabolic pathways” (1252 genes) and “biosynthesis of secondary metabolites” (1995 genes) (**Supplementary Table 6**; **Supplementary Figure 6**). The top 30 enriched KEGG pathways (*P*< 0.05) showed that the most highly enriched KEGG pathway was mainly related to the subcategory “plant hormone signal transduction” (336 genes), “amino sugar and nucleotide sugar metabolism” (145 genes), “Starch and sucrose metabolism” (327 genes), “MAPK signaling pathway” (340 genes), “phenylpropanoid biosynthesis” (196 genes), “pentose and glucuronate interconversions” (111genes), and “flavonoid biosynthesis” (111 genes) (**Supplementary Figure 7**). Additionally, “biosynthesis of amino acids” pathway was also representative in our results and 142 DEGs were enriched in it.

### Analysis of differential gene expression in the phenylpropanoid and flavonoid biosynthesis pathway of *L. kaempferi* callus

To investigate the effects of differential gene expression on metabolic composition and content, we analyzed the expression profile of genes involved in the phenylpropanoid and flavonoid biosynthesis pathways in EC and NEC. According to the results of KEGG enrichment analysis of DEGs, there were 196 genes enriched in phenylpropanoid biosynthesis pathway, in which 107 genes were upregulated while 89 were downregulated in NEC. The 111 genes enriched in flavonoid biosynthesis pathway included 97 upregulated and 14 downregulated genes (**Supplementary Table 7**). Additionally, there were 16 co-regulated genes in the two pathways, among which ten genes were upregulated while six were downregulated. Overall, considerably more genes w ere upregulated in NEC than EC, which is consistent with the outcome of the secondary metabolome analysis (**Supplementary Table 4**).

**Figure 4:**
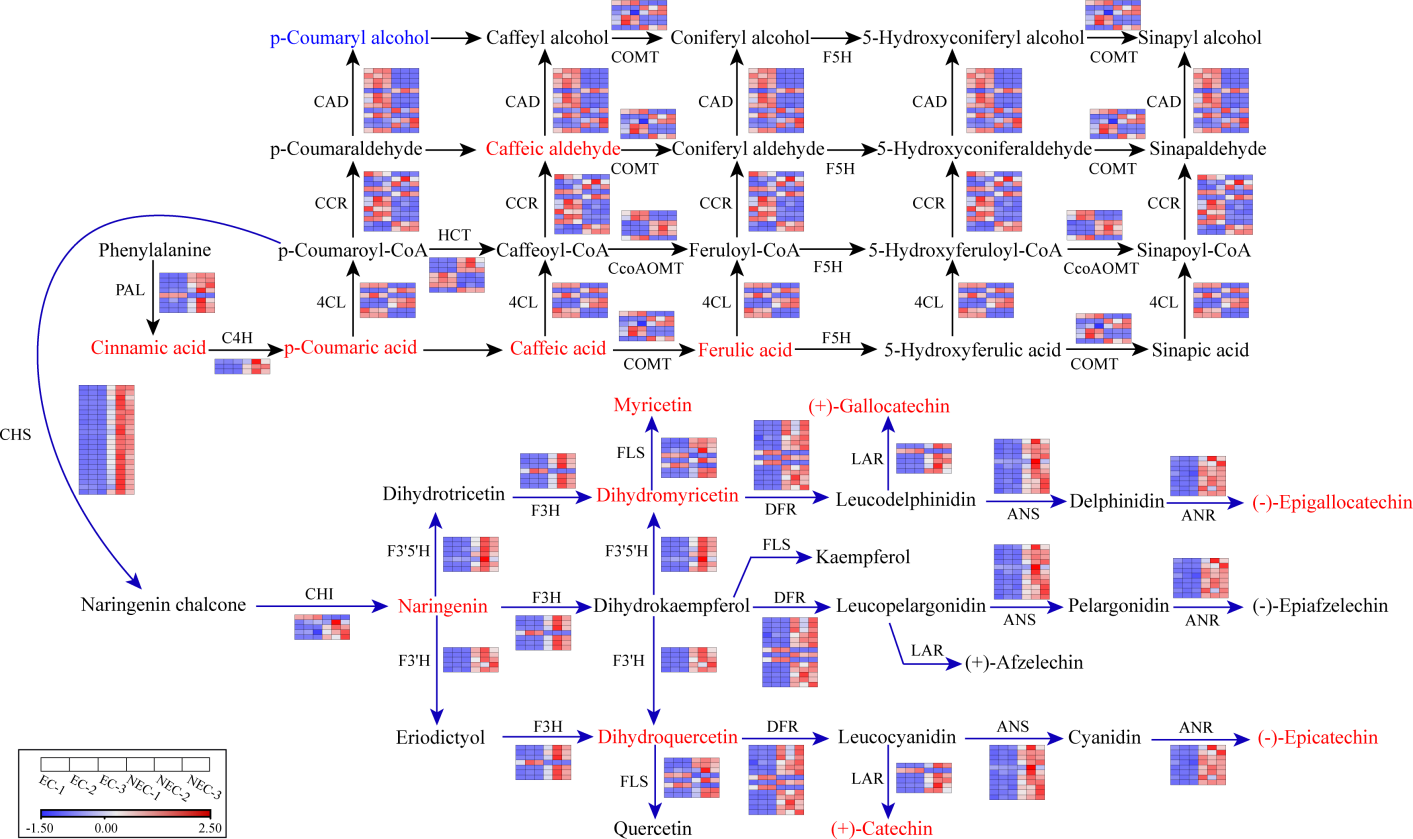
Part of phenylpropanoid and flavonoid metabolic subnetwork with genes and metabolites that constitute the process. Enzyme names and gene expression levels based on FPKM values are indicated at the side of each step. Red and blue blocks represent upregulation and downregulation of gene expression, respectively. Upregulated and downregulated metabolites were indicated in red and blue, respectively. Enzyme abbreviations: PAL, Phenylalanine ammonia-lyase; C4H, Cinnamate 4-hydroxylase; 4CL, 4-Coumarate-CoA ligase; CCR, Cinnamoyl-CoA reductase; HCT: Shikimate O-hydroxycinnamoyltransferase; CCoAOMT: Caffeoyl-CoA O-methyltransferase; COMT: Caffeic acid 3-O-methyltransferase; CHS, Chalcone synthase; CHI, Chalcone isomerase; F3H, Flavonoid 3-hydroxylase; F3′H, Flavonoid 3′ -hydroxylase; F3′5′H, Flavonoid 3’,5’-hydroxylase; FLS, Flavonol synthase; DFR, Dihydroflavonol 4-reductase; LAR, Leucoanthocyanidin reductase; ANS, Anthocyanidin synthase; ANR, Anthocyanidin reductase.

The 16 co-regulated genes involved in both phenylpropanoid and flavonoid biosynthesis pathway were composed of three *C4H* genes (upregulated), six *CcoAOMT* genes (three upregulated and three downregulated), and seven *HCT* genes (four upregulated and three downregulated). Besides these genes, the remaining 97 upregulated genes in phenylpropanoid biosynthesis pathway included seven *PAL* genes, three *4CL* genes, three *COMT* genes, four *CAD* genes, and five *CCR* genes. The 83 downregulated genes in phenylpropanoid biosynthesis pathway included one *PAL* gene, four *4CL* genes, three *COMT* genes, nine *CAD* genes, and seven *CCR* genes. For flavonoid biosynthesis pathway, the remaining 87 upregulated genes included 22 *CHS* genes, four *CHI* genes, five *F3H* genes, five *F3’H* genes, seven *F3’5’H* genes, 12 *DFR* genes, six *FLS* genes, eight *ANS* genes, 14 *ANR* genes, and five *LAR* genes. The eight downregulated genes included one *CHI* gene, one *F3H* gene, two *DFR* genes, two *FLS* genes, and one *LAR* gene. Based on the differential gene expression profile, we reconstructed the phenylpropanoid and flavonoid biosynthesis pathways in *L. kaempferi* callus (**Figure 4**).

### qRT-PCR validation of differential expression

**Figure 5:**
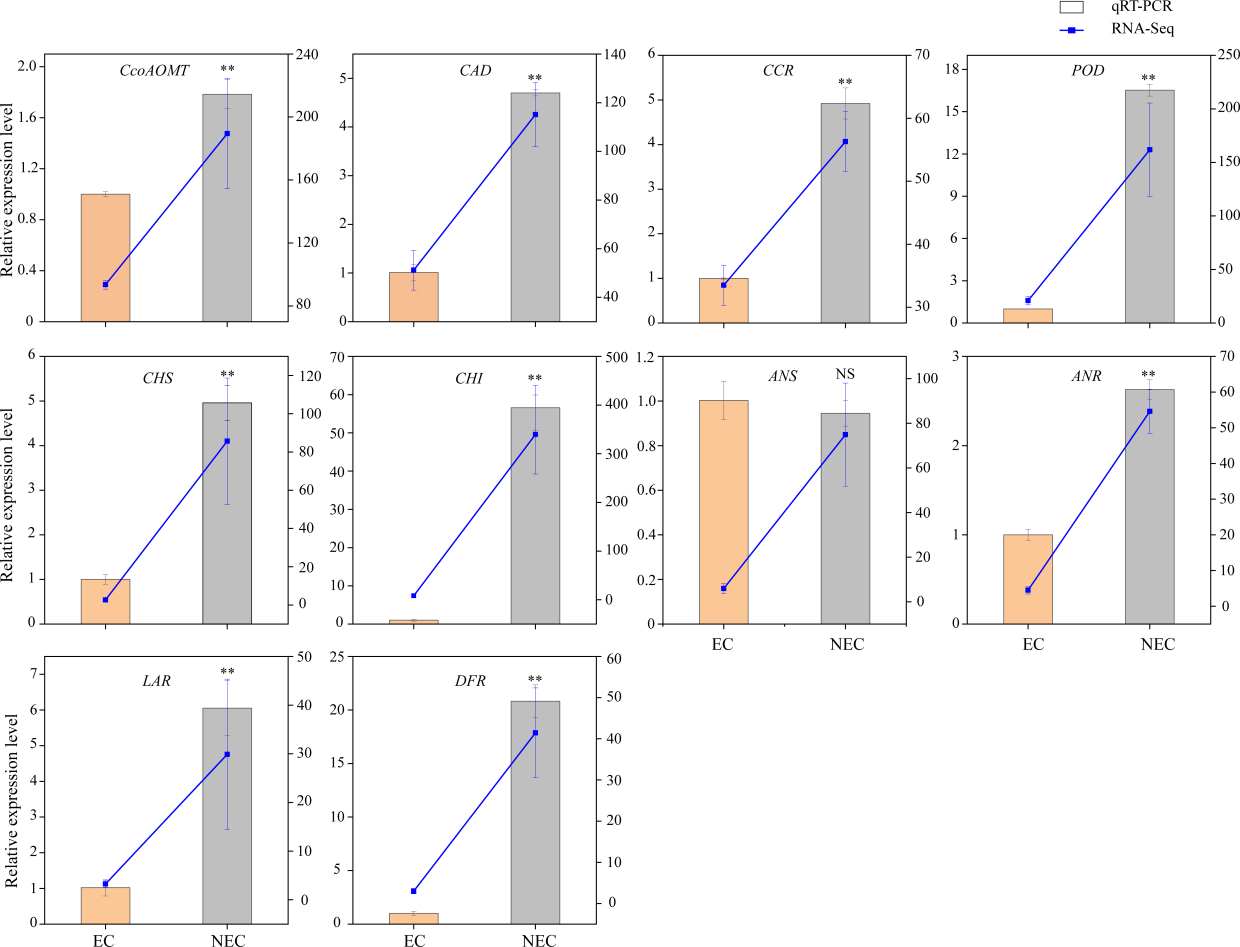
Comparison of expression profiles of ten selected genes in *L. keampferi* calluses as measured by quantitative real-time polymerase chain reaction (qRT-PCR) and RNA-seq. Columns represent gene expression determined by qRT-PCR (left y-axis), while lines represent gene expression determined by RNA-seq in FPKM values (right y-axis). The error bars represent the SD from three biological replicates. *, statistical significance at *P* < 0.05; **, statistical significance at *P* < 0.01; NS, not significant.

To verify the accuracy of the transcriptome data, the expression levels of ten candidate genes related to phenolic acid and flavonoid biosynthesis were determined using qRT-PCR. The results showed that the expression levels of nine out of the ten candidate genes differed between EC and NEC, and showed expression patterns similar to those of the transcriptome data (**Figure 5**). Therefore, our RNA-seq and qRT-PCR analysis results showed high reliability and can be used for further analysis.

### Correlation analysis between transcriptome and metabolome data

**Figure 6:**
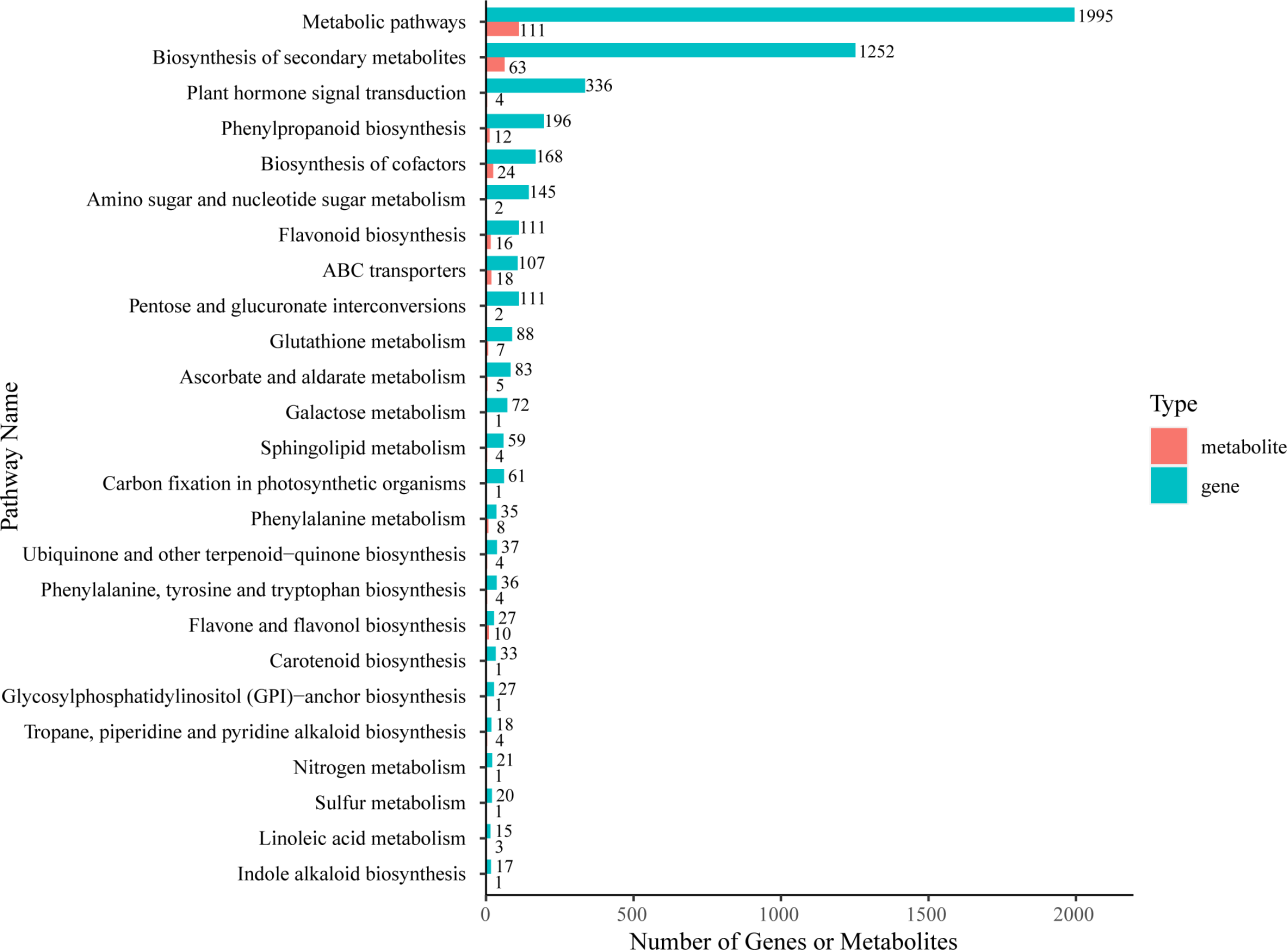
KEGG enrichment analysis of DEGs (green column) and DAMs (red column) that were enriched in the same pathway.

Firstly, we used KEGG enrichment analysis to screen out DAMs and DEGs that could be enriched in the same pathway (**Figure 6**; **Supplementary Table 8**). The results showed that the most DEGs and DAMs were enriched in “metabolic pathways” (1995 genes and 111 metabolites) and “biosynthesis of secondary metabolites” (1252 genes and 63 metabolites). Additionally, there are four KEGG pathways enriched with more DEGs and DAMs than other pathways– “biosynthesis of cofactors” (168 DEGs and 24 DAMs), “ABC transporters” (107 DEGs and 18 DAMs), “phenylpropanoid biosynthesis” (196 DEGs and 12 DAMs), and “flavonoid biosynthesis” (111 DEGs and 16 DAMs).

Pearson correlation analysis was performed to better understand the correlation relationship between DEGs and DAMs which were enriched in the same metabolic pathway. We selected 62 DEGs and 109 DEGs on the major branches in flavonoid biosynthesis and phenylpropanoid biosynthesis pathway, respectively, for this analysis.

The screening criteria were a *P*-value < 0.01 and a PCC-value > 0.9. In phenylpropanoid biosynthesis pathway, 53 DEGs were significantly correlated with 12 DAMs, resulting in 326 related pairs (159 positive and 167 negative pairs) (**Supplementary Table 9**). In flavonoid biosynthesis pathway, 94 DEGs were correlated with 16 DAMs and a total of 1137 related pairs (912 positive and 225 negative pairs) were found. Among them, it is ubiquitous that a single gene was regulated by multiple metabolites, or a single metabolite was regulated by multiple genes. For example, in phenylpropanoid biosynthesis pathway, the content of mws2213 (cinnamic acid) and pme1439 (p-Coumaric acid) was significantly correlated with the expression levels of 38 DEGs (18 positively correlated and 20 negatively correlated) and 30 DEGs (13 positively correlated and 17 negatively correlated), respectively. The expression level of the gene Lk17760 annotated as c*affeoyl-CoA O-methyltransferase* (*CcoAOMT*) was significantly positively correlated with the accumulation of HJN003 (1-O-sinapoyl-D-glucose), mws0906 (coniferin), mws2213 (Cinnamic acid), pmb0751 (trans-5-O-(p-Coumaroyl) shikimate), pme0021 (L-Phenylalanine), and pme2993 (scopoletin(7-Hydroxy-5-methoxycoumarin). Similarly, in flavonoid biosynthesis pathway, the accumulation of metabolites such as mws0044 (dihydroquercetin) and mws0054 (catechin*) were significantly correlated with the expression levels of 86 DEGs (76 positively correlated and 20 negatively correlated) and 78 DEGs (69 positively correlated and 9 negatively correlated), respectively. The expression level of Lk17613, which was annotated as *naringenin 3-dioxygenase* (*F3H*), showed significant positive correlation relationship with the accumulation with mws0032 (myricetin), mws0044 (dihydroquercetin), mws0049 (gallocatechin*), mws0054 (catechin*), mws0744 (dihydromyricetin), mws0914 (pinobanksin), mws1173 (garbanzol), and mws2118 (phlorizin) while it was negatively correlated with MWSHY0089 (sakuranetin) and pmb0751 (trans-5-O-(p-Coumaroyl) shikimate) (**Supplementary Table 10**).

Additionally, we found that the expression levels of the 14 co-regulated DEGs involved in both phenylpropanoid and flavonoid biosynthesis pathways were all significantly correlated with the accumulation of the DAMs enriched in the two pathways. For example, Lk17760 (annotated as *caffeoyl-CoA O-methyltransferase*) were significantly correlated with six metabolites (six positively correlated) in phenylpropanoid biosynthesis pathway and nine metabolites in flavonoid biosynthesis pathway (eight positively correlated and one negatively correlated). Therefore, these genes may play an important role in the accumulation of metabolites in the two pathways (**Table 3**).

**Table 3:** Co-regulated DEGs and their relationship with DAMs in phenylpropanoid and flavonoid biosynthesis pathway.

GeneID	Annotation	Regulated (EC vs NEC)	Phenylpropanoid biosynthesis			Flavonoid biosynthesis		
			CM	P	N	CM	P	N
Lk00868	shikimate O-hydroxycinnamoyltransferase [EC:2.3.1.133]	up	2	2	0	2	2	0
Lk09014	caffeoyl-CoA O-methyltransferase [EC:2.1.1.104]	down	8	2	6	12	2	10
Lk09659	caffeoyl-CoA O-methyltransferase [EC:2.1.1.104]	down	8	2	6	12	2	10
Lk12611	shikimate O-hydroxycinnamoyltransferase [EC:2.3.1.133]	down	7	1	6	10	9	1
Lk12671	caffeoyl-CoA O-methyltransferase [EC:2.1.1.104]	down	8	1	7	7	1	6
Lk17759	caffeoyl-CoA O-methyltransferase [EC:2.1.1.104]	up	9	2	7	16	14	2
Lk17760	caffeoyl-CoA O-methyltransferase [EC:2.1.1.104]	up	6	6	0	9	8	1
Lk18448	trans-cinnamate 4-monooxygenase [EC:1.14.14.91]	up	7	1	6	10	9	1
Lk21778	shikimate O-hydroxycinnamoyltransferase [EC:2.3.1.133]	down	8	2	6	12	1	11
Lk29217	caffeoyl-CoA O-methyltransferase [EC:2.1.1.104]	up	8	2	6	14	13	1
Lk31540	shikimate O-hydroxycinnamoyltransferase [EC:2.3.1.133]	up	5	4	1	11	9	2
Lk37551	shikimate O-hydroxycinnamoyltransferase [EC:2.3.1.133]	down	4	1	3	9	2	7
Lk38153	trans-cinnamate 4-monooxygenase [EC:1.14.14.91]	up	8	6	2	13	11	2
Lk44139	shikimate O-hydroxycinnamoyltransferase [EC:2.3.1.133]	up	6	2	4	9	7	2

CM, the number of metabolites which were significantly correlated with the DEGs. P and N, the number of metabolites which were positively and negatively correlated with the DEGs, respectively.

### Differential expression analysis of genes related to plant hormone signaling pathway

Based on the results of KEGG enrichment analysis of DEGs and DAMs above, we found that many DEGs and DAMs were enriched in “plant hormone biosynthesis” and “plant hormone signal transduction” pathways. 336 DEGs and four DAMs were enriched in “plant hormone signal transduction” pathway. In plant hormone biosynthesis, a total of 212 DEGs and 18 DAMs were involved in jasmonic acid (JA) (62 DEGs and three DAMs), ethylene (ETH) (59 DEGs and four DAMs), cytokinine (CTK) (38 DEGs and three DAMs), and IAA (53 DEGs and eight DAMs) biosynthesis.

**Figure 7:**
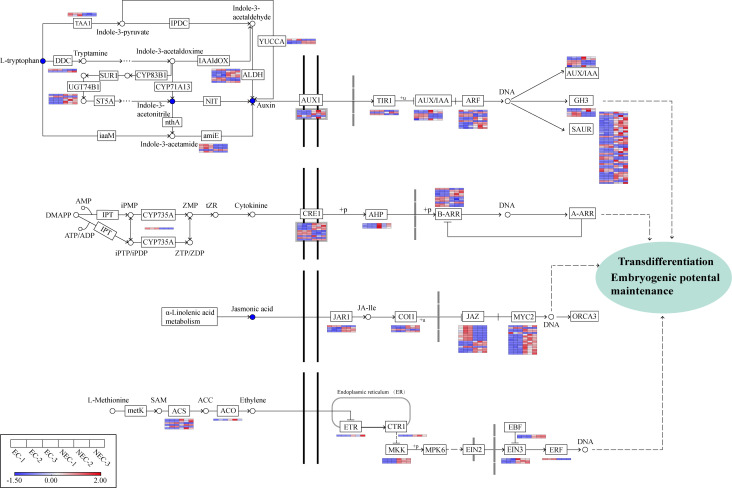
Analysis of DEGs involved in plant hormone synthesis and signal transduction pathways. Gene expression levels based on FPKM values are indicated at the side of each step. Red and blue blocks represent upregulation and downregulation of gene expression, respectively. Enzyme abbreviations involved in plant hormone synthesis: TAA1, L-tryptophan—pyruvate aminotransferase; DDC, L-tryptophan decarboxylase; UGT74B1, N-hydroxythioamide S-beta-glucosyltransferase; ALDH, aldehyde dehydrogenase; amiE, amidase; YUCCA, indole-3-pyruvate monooxygenase; CYP735A, cytokinin trans-hydroxylase; ACS, 1-aminocyclopropane-1-carboxylate synthase; ACO, aminocyclopropanecarboxylate oxidase.

In the JA pathway, the content of JA in NEC was found to be higher than that in EC (**Supplementary Table 4**). There were 41 genes related to JA signal transduction, in which 27 genes were upregulated while 14 were downregulated. The 27 upregulated genes in NEC included three genes encoding jasmonic acid-amido synthetase (JAR), six genes encoding jasmonate ZIM domain-containing protein (JAZ), three genes encoding coronatine-insensitive protein 1 (COI1), and 15 genes encoding transcription factor MYC2 (MYC2). The 14 downregulated genes contained one *JAR* gene, ten *JAZ* genes, one *COI1* gene, and two *MYC2* genes (**Figure 7**).

L-Methionine to S-Adenosyl-L-methionine (SAM), SAM to 1-aminocyclopropane-1-carboxylic acid (ACC), and ACC to ETH are three key conversions in the ETH biosynthesis pathway (Zhang et al., 2022). In this study, four genes encoding 1-aminocyclopropane-1-carboxylate synthase (ACS) and one gene encoding aminocyclopropanecarboxylate oxidase (ACO) involved in the latter two catalytic processes were found to be upregulated in NEC while only two ACS genes were downregulated in NEC. 11 genes were enriched in the ETH signal transduction pathway, among which one gene encoding ethylene receptor (ETR), one gene encoding serine/threonine-protein kinase CTR1 (CTR1), three genes encoding mitogen-activated protein kinase kinase 4/5 (MKK4/5), three genes encoding ethylene-insensitive protein 3 (EIN3), and two genes encoding EIN3-binding F-box protein (EBF) were upregulated in NEC, whereas only one gene encoding ethylene-responsive transcription factor 1 (ERF1) was downregulated in NEC.

In the CTK biosynthesis pathway, one gene encoding the key enzyme cytokinin trans-hydroxylase (CYP735A) in CTK biosynthesis showed a higher expression in EC than NEC. Some genes related to CTK signal transduction such as six genes encoding cytokinin receptor 1 (CRE1), three genes encoding histidine-containing phosphotransfer protein (AHP), and five *ARR-B* gene family members were upregulated in NEC, whereas four *CRE1* genes and six *ARR-B* genes were downregulated in NEC.

In our results, the content of three key metabolites in IAA biosynthesis pathway―L-tryptophan (mws0282), indole-3-acetonitrile (pmb0819), and indole 3-acetic acid (IAA) (pme1651) in EC was higher than that in NEC. Likewise, some genes involved in IAA biosynthesis and signal transduction pathways were differentially expressed between EC and NEC. Among the 53 genes related to IAA biosynthesis, three genes encoding aldehyde dehydrogenase (ALDH), four genes encoding amidase (amiE), one gene encoding aromatic-L-amino-acid/L-tryptophan decarboxylase (DDC), one gene encoding indole-3-pyruvate monooxygenase (YUC), one gene encoding N-hydroxythioamide S-beta-glucosyltransferase (UGT74B1), two genes encoding L-tryptophan—pyruvate aminotransferase (TAA1) were downregulated in NEC, whereas five *ALDH* genes, one *amiE* gene, one *DDC* gene, two *YUC* genes, and five *UGT74B1* genes were upregulated in NEC. We screened out 72 DEGs associated with IAA signal transduction, including 48 upregulated and 24 downregulated genes in NEC, and the genes encoding SAUR family protein (SAUR) accounted for the largest proportion (42 genes/58.3%) of all these genes, among which 31 *SAUR* genes were upregulated while 11 were downregulated in NEC. Besides, the expression of one gene encoding auxin influx carrier (AUX1), two genes encoding transport inhibitor response 1 (TIR1), three genes encoding auxin-responsive protein IAA (AUX/IAA), four genes encoding auxin response factor (ARF), three genes encoding auxin responsive GH3 gene family (GH3) was downregulated in NEC, while four *AUX1* genes, one *TIR1* gene, three *AUX/IAA* genes, seven *ARF* genes, and two *GH3* genes were upregulated in NEC (**Figure 7**).

### Analysis of genes encoding TFs

**Figure 8:**
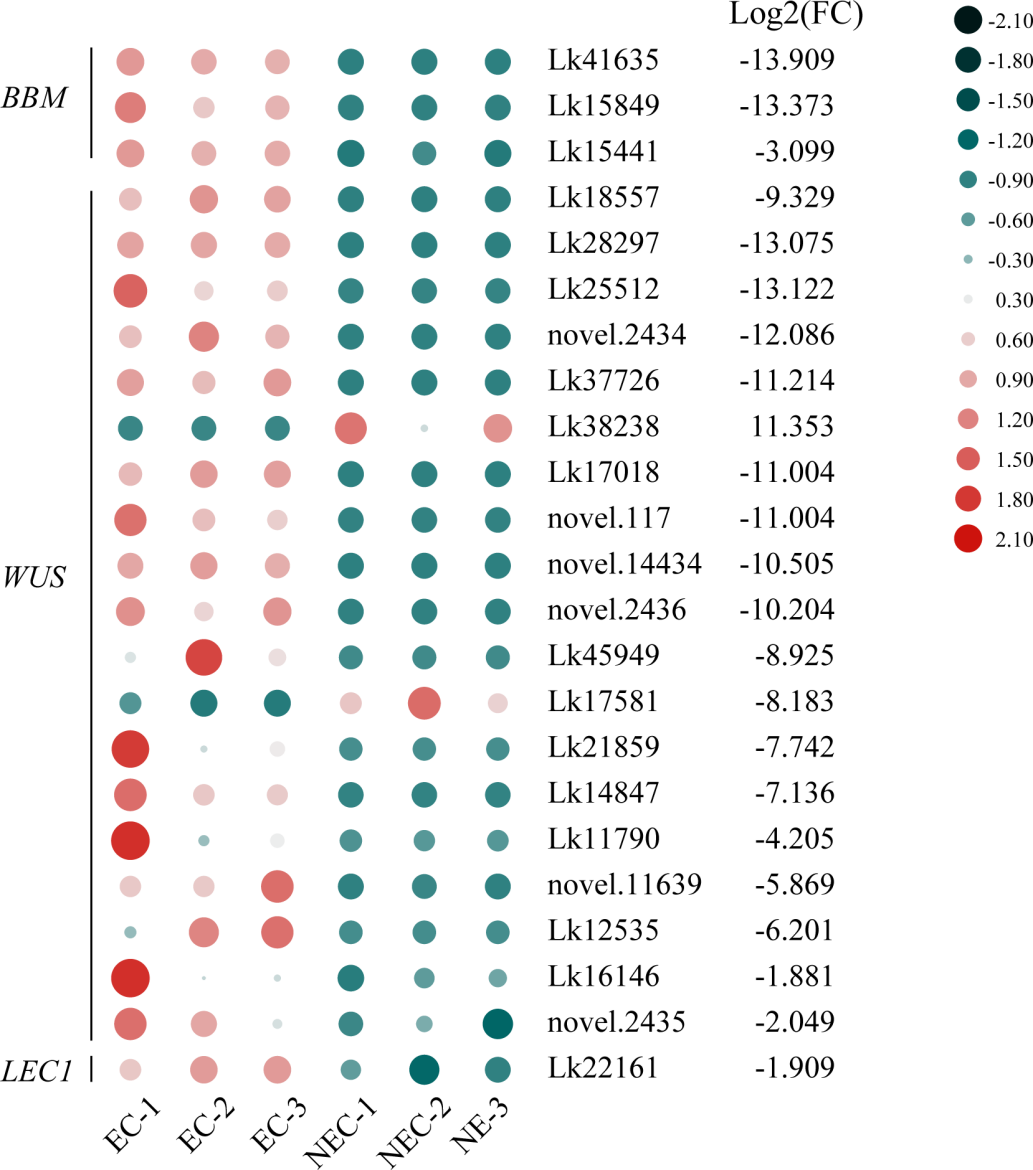
Expression patterns of totipotency-related transcription factor (TF) genes between EC and NEC in *L. kaempferi*. Gene expression levels were calculated based on FPKM values. Red and green dots represent upregulation and downregulation of gene expression, respectively.

TFs are of great importance in regulating various development-related processes as well as secondary metabolite production in plants. In this study, 3,066 genes were recognized as TF genes and belonged to 90 TF families. Among all the TF genes, 787 genes were differentially expressed between EC and NEC and can be furtherly classified into 73 TF families (**Supplementary Table 11**), among which 405 TF genes were upregulated while 382 were downregulated in NEC compared to EC. The TF families with the most genes which were differentially expressed between the two groups were ERF, bHLH, MYB, NAC, and LOB, with 64, 58, 46, 37, and 32 genes included, respectively (237 in total). Among these five major TF families, the number of all TF genes upregulated in NEC were greater than that were downregulated. Specifically, 134 TF genes were upregulated while 103 were downregulated in NEC, in which 37 *ERFs*, 32 *bHLHs*, 29 *MYBs*, 18 *NACs*, and 18 *LOBs* were found to be upregulated while 27 *ERFs*, 26 *bHLHs*, 17 *MYBs*, 19 *NACs* and 14 *LOBs* were downregulated in NEC. Besides, totipotency related TF genes showed contrasting expression levels between EC and NEC. Three *BBM* genes, 17 *WUS* genes, and one *LEC1* gene were upregulated while only two *WUS* genes were downregulated in NEC compared to EC (**Figure 8**).

### Possible transcriptional regulatory network involved in phenolic acid and flavonoid biosynthesis

To more accurately identify the TFs that may play important roles in regulating the expression of functional genes involved in both phenolic acid and flavonoid biosynthesis, we used the PlantTFDB online database to predict the TFs that potentially regulated the co-regulated structural genes enriched in both phenylpropanoid and flavonoid biosynthesis pathways. The eight upregulated DEGs were selected for this analysis as the expression levels of these genes were more directly associated with the accumulation of the key metabolites (**Table 3**). Unfortunately, we did not find the TFs which may potentially regulate *CcoAOMT* (Lk29217), so we kept the remaining seven genes for further analysis. Based on the targeted relationship, a total of 34 differentially expressed TF genes between EC and NEC were identified as candidate regulatory genes (**Table 4**).

**Table 4:** The expression and annotation of candidate TFs regulating structural genes involved in flavonoid and phenolic acid biosynthesis.

Gene ID	Family	Expression level (mean ± SD)		Differential expression		Node degree	Description
		EC	NEC	log2(FC)	*P*-value		
Lk42000	MYB	0 ± 0	3.92 ± 3.73	10.06	5.25E-09	3	myb domain protein 61
Lk32611	MYB	3.29 ± 0.32	10.66 ± 4.95	1.98	2.16E-08	4	myb domain protein 3
Lk32612	MYB-related	1.59 ± 0.44	0.01 ± 0.02	-6.4	9.09E-07	4	myb domain protein 3
Lk35445	MYB	0.07 ± 0.09	6.87 ± 2.05	6.87	4.65E-20	4	myb domain protein 3
Lk36906	MYB-related	0.13 ± 0.15	1.74 ± 0.42	4.03	2.09E-08	4	myb domain protein 3
Lk23436	AP2/ERF-ERF	1.48 ± 0.45	9.33 ± 1.11	2.93	1.53E-23	3	ethylene responsive element binding factor 3
Lk33289	AP2/ERF-ERF	25.69 ± 0.26	0 ± 0	-11.01	2.46E-20	3	ethylene responsive element binding factor 3
Lk31643	AP2/ERF-ERF	4.08 ± 0.3	18.73 ± 7.04	2.46	2.36E-17	3	ERF family protein
Lk45868	AP2/ERF-ERF	32.33 ± 4.28	115.28 ± 18.9	2.1	2.91E-35	2	related to AP2 4
Lk09187	BBR-BPC	20.56 ± 0.99	6.79 ± 0.96	-1.33	8.49E-17	5	basic pentacysteine1
Lk14515	AP2/ERF-ERF	1.87 ± 0.4	0.03 ± 0.05	-5.9	1.82E-07	2	ERF family protein
Lk38872	TCP	80.83 ± 2.13	16.7 ± 0.98	-2	1.09E-84	2	TCP family protein
Lk18449	C3H	28.16 ± 1.01	4.85 ± 2.21	-2.29	3.83E-16	2	C3H family protein
Lk42876	AP2/ERF-ERF	2.33 ± 0.48	9.68 ± 4.23	2.31	6.11E-13	2	ethylene responsive element binding factor 4
Lk28653	AP2/ERF-ERF	144.76 ± 22.12	550.76 ± 30.77	2.2	1.26E-46	1	ethylene-responsive element binding protein
Lk31637	AP2/ERF-ERF	5.89 ± 0.83	54.44 ± 47.5	3.47	1.85E-05	1	ethylene-responsive element binding protein
Lk31642	AP2/ERF-ERF	18.98 ± 3.8	61.9 ± 38.48	1.95	2.51E-03	1	ethylene-responsive element binding protein
Lk27823	AP2/ERF-ERF	9.1 ± 0.41	3.66 ± 0.2	-1.04	3.43E-16	2	ethylene response factor 1
Lk09485	AP2/ERF-ERF	15.48 ± 1.61	1.89 ± 0.86	-2.78	1.61E-23	3	cytokinin response factor 4
Lk19358	MYB	0.44 ± 0.1	2.73 ± 0.74	2.89	2.92E-09	3	myb domain protein 4
Lk34626	MYB	1.6 ± 0.63	20.9 ± 14.62	4.02	1.81E-14	3	myb domain protein 4
Lk36102	MYB	0 ± 0	1.26 ± 0.46	7.38	4.76E-08	3	myb domain protein 4
Lk37647	MYB	0.74 ± 0.41	4.62 ± 0.79	2.94	3.38E-11	3	myb domain protein 4
Lk37651	MYB	0.37 ± 0.11	1.59 ± 0.47	2.36	9.60E-06	3	myb domain protein 4
Lk02128	MYB	3.54 ± 0.34	0.28 ± 0.26	-3.42	1.20E-07	2	myb domain protein 43
Lk07979	AP2/ERF-ERF	0.53 ± 0.1	22.67 ± 6.51	5.69	3.38E-63	2	ERF family protein
Lk29727	AP2/ERF-ERF	6.62 ± 1.12	0 ± 0	-9.49	5.86E-15	2	ERF family protein
Lk20608	AP2/ERF-ERF	2.50 ± 0.43	0.3 ± 0.03	-2.79	6.56E-15	3	ERF family protein
Lk10480	AP2/ERF-ERF	6.98 ± 2.08	12.88 ± 2.46	1.15	1.12E-04	4	erf domain protein 9
Lk23435	AP2/ERF-ERF	8.78 ± 1.27	19.31 ± 6.74	1.39	2.34E-07	4	erf domain protein 9
Lk28118	AP2/ERF-ERF	30.48 ± 0.73	3.02 ± 1.27	-3.04	5.42E-23	4	erf domain protein 9
Lk33170	AP2/ERF-ERF	68.19 ± 2.47	19.33 ± 1.54	-1.54	1.79E-30	4	erf domain protein 9
Lk37167	C2C2-Dof	3.27 ± 0.45	196.71 ± 19.3	6.18	0	2	OBF binding protein 4
Lk44672	LOB	6.74 ± 2.03	0 ± 0	-9.69	2.90E-15	4	LBD family protein

FC, fold change. The expression levels of genes were calculated based on FPKM values.

**Figure 9:**
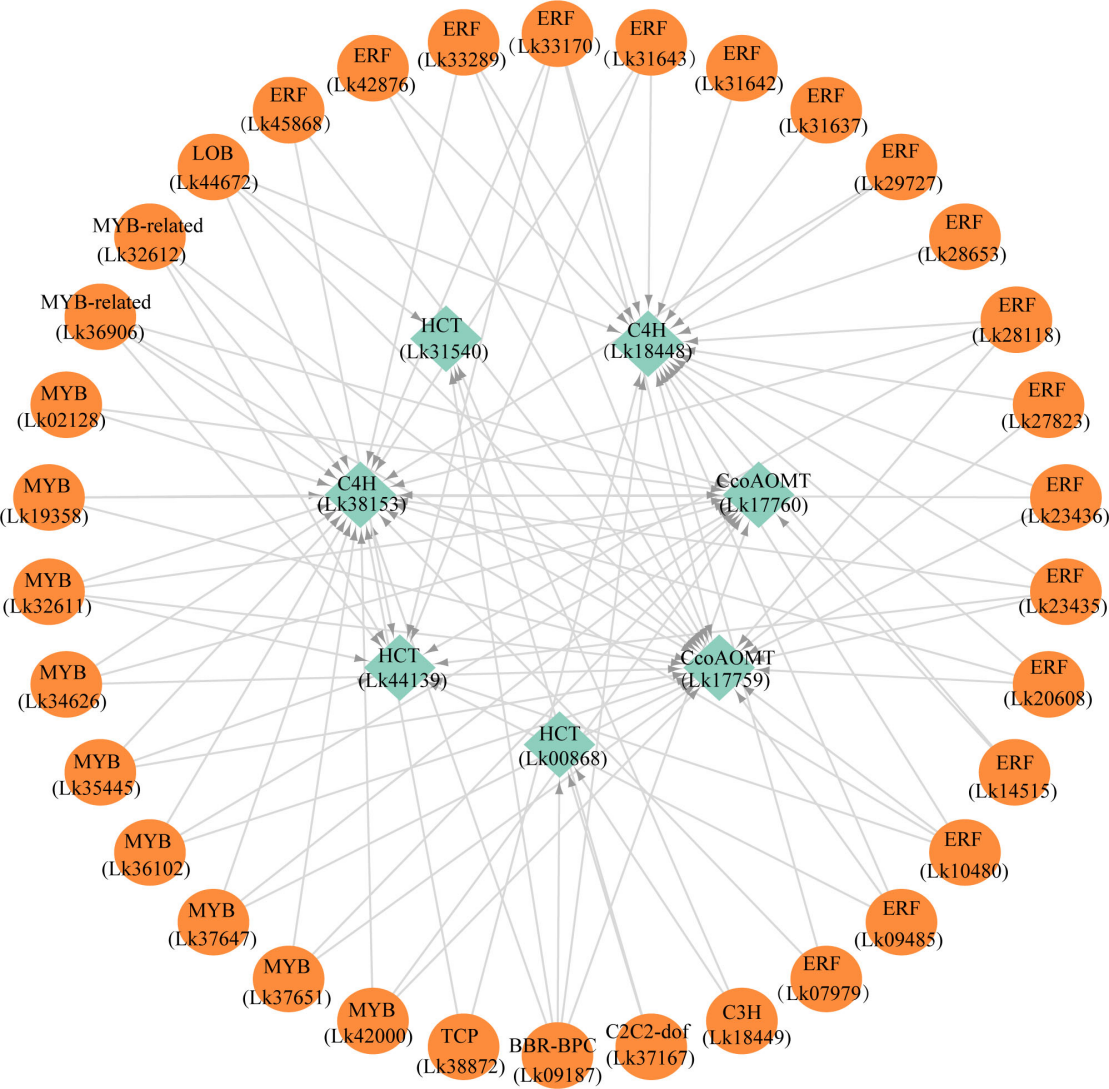
The potential regulatory network between TFs and key structural genes involved in phenylpropanoid and flavonoid biosynthesis pathways in *L. kaempferi* callus.

The regulatory relationship between candidate TFs and the seven target genes was shown in **Figure 9**. The node degree of TFs was defined as the number of target genes one TF may regulate, which could represent the importance of TFs in the metabolic pathway (Xiao et al., 2021). The results showed that each TF had several target genes with a node degree ranging from 1 to 5 (**Table 4**; **Figure 9**). The node degree of BBR-BPC (Lk09187) was the highest among all the 34 TFs, with a value of five. Four ERFs including Lk10480, Lk23435, Lk28118, and Lk33170, LOB (Lk44672), two MYBs including Lk32611 and Lk35445, and two MYB-related TFs including Lk32612 and Lk36906 had the second highest node degree, with a value of four. The four ERFs all showed target relationship with one *CcoAOMT* gene (Lk17759), two *C4H* genes (Lk18448 and Lk38153), and one *HCT* gene (Lk44139). The two MYBs and two MYB-related TFs all showed target relationship with two *CcoAOMT* genes (Lk17759 and Lk17760), one *C4H* gene (Lk38153), and one *HCT* gene (Lk44139). Additionally, 11 TFs including five ERFs and six MYBs had a relatively high node degree, with a value of three. Thus, these TFs were considered as important TFs, which may be crucial in regulating the expression of key structural genes involved in the flavonoid and phenylpropanoid biosynthesis pathways and thus leading to the differential accumulation of flavonoids and phenolic acids between EC and NEC.

**Figure 10:**
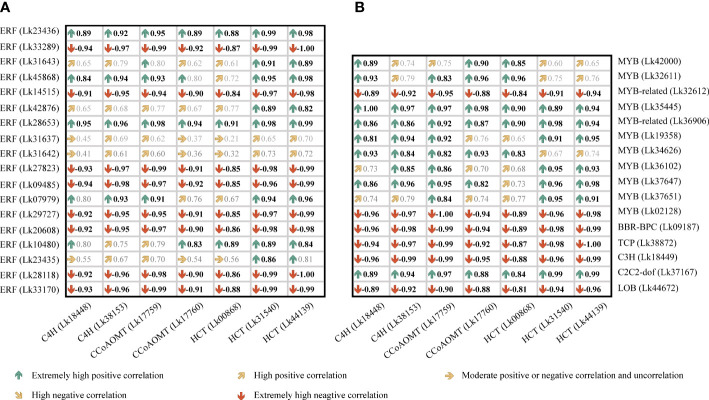
Correlation analysis of the expression levels between candidate TF genes and key structural genes involved in phenylpropanoid and flavonoid biosynthesis in *L. kaempferi* callus. **(A)** Correlation relationship between ERF genes and key structural genes. **(B)** Correlation relationship between other TF genes and key structural genes. Bold black indicates significant correlation at *P*< 0.05.

To better figure out the relationship between these candidate TF genes and the co-regulated structural genes, a Pearson correlation analysis was conducted based on the FPKM values of these genes (**Figure 10**). Although BBR-BPC and LOB had the highest node degrees among all TFs, their transcription levels all showed negative correlations with their target genes. For ERF members, the nine ERFs showed relatively high node degrees, but only Lk23436 was significantly positively correlated with all its target genes including two *C4H* genes (Lk18448 and Lk 38153) and one *CcoAOMT* gene (Lk17759). For MYB and MYB-related members, Lk34626 and Lk35445 were highly corelated with their target genes, whereas only Lk34626 showed high transcription level in NEC (FPKM= 20.90), which was approximately 13 times higher than that in EC. It’s worth mentioning that two candidate TFs (node degree= 2), C2C2-dof (Lk37167) and ERF (Lk45868), showed high transcription levels in NEC (FPKM= 196.71 and 115.28, respectively) and showed significantly positive correlations with their target genes. To sum up, we speculated that ERF (Lk23436 and Lk45868), MYB (Lk34626), and C2C2-dof (Lk37617) were the most important TFs in regulating phenolic acid and flavonoid biosynthesis.

### Regulation module governing the differential accumulation of phenolic acids and flavonoids between EC and NEC

**Figure 11:**
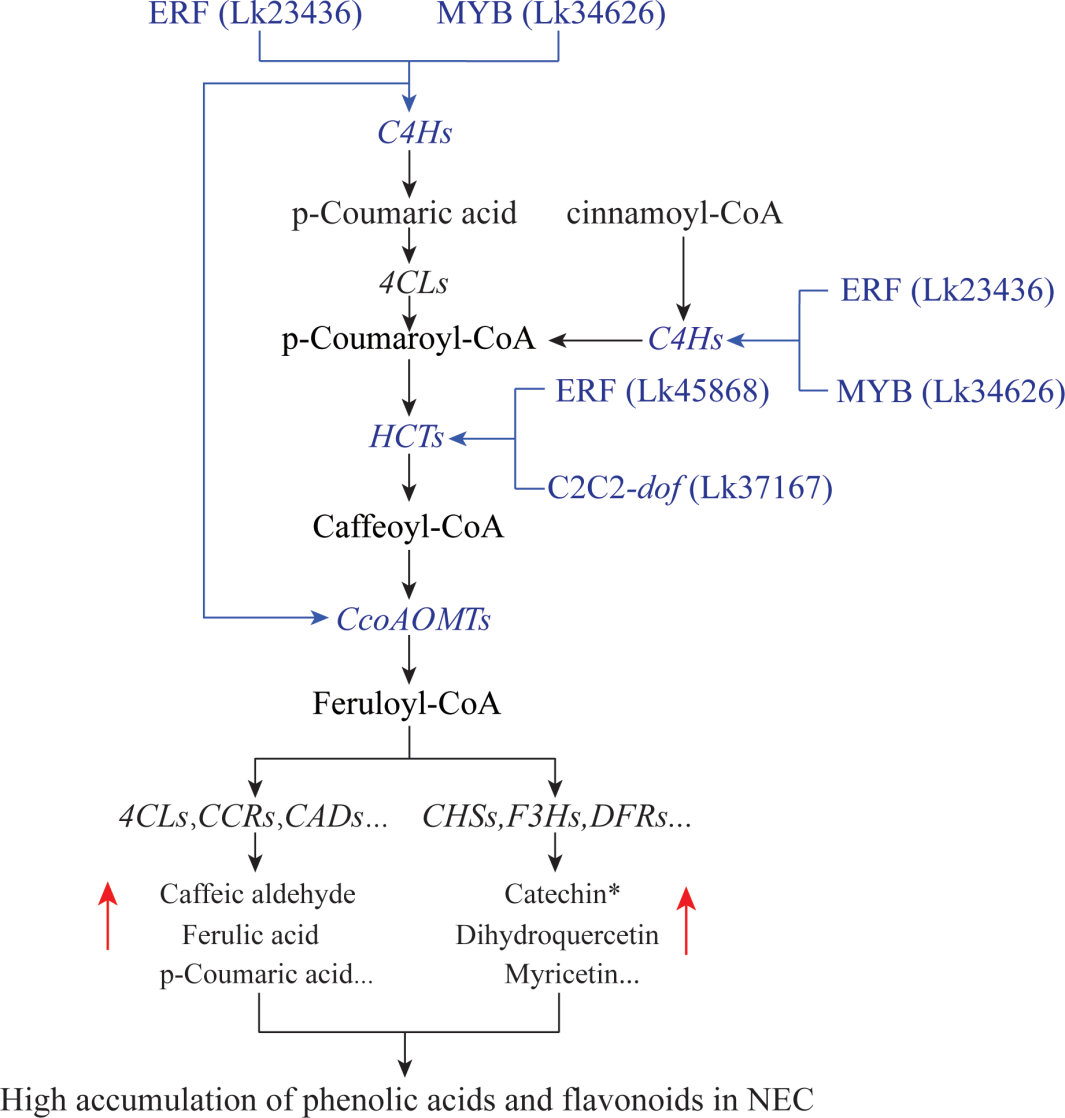
Potential transcriptional regulatory module governing the differential accumulation of flavonoids and phenolic acids between EC and NEC. Possible regulatory modules were highlighted in blue.

According to the targeted relationship, the correlations between the TFs and the key structural genes based on the expression profile as well as the differential accumulation of metabolites in the phenylpropanoid and flavonoid biosynthesis pathways between groups, we preliminary proposed the transcriptional regulatory mechanisms underlying flavonoid and phenolic acid biosynthesis in *L. kaempferi* calluses (**Figure 11**). During SE induction, two types of callus including EC and NEC may be generated. They differed significantly in secondary metabolic activities and several TFs were involved in these metabolic processes. The transcription levels of ERF (Lk23436) and MYB (Lk34626) were significantly increased in NEC and they could bind *C4H* genes to activate their expression, contributing to the increase of p-Coumaric acid. The two substances including p-Coumaric acid and cinnamoyl-CoA were used as the substrates for further reactions. ERF (Lk45868) and C2C2-dof (Lk37617) bound *HCT* genes to promote the conversion of p-Coumaroyl-CoA to caffeoyl-CoA. Then, ERF (Lk23436) and MYB (Lk34626) furtherly enhanced the expression of *CcoAOMT* genes, leading to the conversion of caffeoyl-CoA to feruloyl-CoA. By integrating other structural genes such as *4CL*, *CCR*, and *CAD* genes in phenylpropanoid biosynthesis pathway or *CHS, F3H*, and *DFR* genes in flavonoid biosynthesis pathway, the contents of phenolic acids and flavonoids were ultimately increased in NEC.

## Discussion

As in many conifers, rooting of cuttings or seed propagation cannot satisfy the needs of mass reproduction of new lines with desired characteristics in *L. kaempferi*. SE is a flexible and efficient tool in providing enough uniform genetically improved material (Gautier et al., 2019), but the instability of EC induction has limited the application of SE in many plants. It has demonstrated that SE induction was a complex process regulated by a network of hundreds of genes (Mordhorst et al., 1997; Wang et al., 2020; Ávila et al., 2022). As metabolites are the end product of gene expression, we considered that many metabolic processes must be involved when EC and NEC were formed. However, metabolic changes between EC and NEC were rarely emphasized in conifers in previous studies. In this study, an integrated transcriptomic and metabolomic profiling was performed to identify genes/metabolic pathways that are differentially represented between groups and provides important insights into the molecular mechanisms underlying embryogenic potential maintenance and the differential accumulation of key metabolites between EC and NEC during SE induction in this species, thus leading to a better understanding of SE induction process that resulted in plant cell totipotency as well as the global association between gene expression and metabolite accumulation.

### Essential role of totipotency-related TFs in EC

The obvious differences in color, texture, morphology or histology between EC and NEC of different species have been reported in several studies (Nagmani and Bonga, 1985; Maadon et al., 2016; Bravo et al., 2017; Gautier et al., 2019; Xue et al., 2022), but the most significant difference between EC and NEC is that EC cells have the potential to acquire totipotency, which can furtherly regenerate into a complete organism (Condic, 2014; Horstman et al., 2017; Li et al., 2022b). A number of plant TFs have been identified that can covert somatic cells into embryogenic, totipotent cells, in which BBM, WUS, and LEC1 have been highlighted in many studies (Su et al., 2009; Horstman et al., 2017; Su et al., 2021; Wang et al., 2022a). In this study, the expression levels of most of these TF genes were higher in EC than those in NEC (**Figure 8**), which may indicate the important roles of these TFs in cell dedifferentiation resulting in the totipotency fate determination of somatic plant cells during EC induction process in *L. kaempferi*. These results were consistent with the findings in *Pinus radiata* (Bravo et al., 2017). It’s thought that the initiation of SE requires an inductive signal that causes a somatic cell to change its fate and become totipotent (Su et al., 2021). In Arabidopsis, exogenous 2,4-D could evoke the increased transcript levels of totipotency-related TFs including BBM, WUS, LEC1, and LEC2 (Su et al., 2009; Wang et al., 2020). The changes of *BBM* and *LEC2* may be directly induced by auxin response factor (ARF) TFs because of the presence of putative ARF-binding motifs in the promoter regions of *BBM* and *LEC2* (Grzybkowska et al., 2020; Wang et al., 2020). In our results, several *ARF* genes also showed higher expression levels in EC (**Figure 7**). The expression of these genes may contribute to the high embryogenic potential of EC and endow part of EC cells with totipotency. Endogenous IAA levels are of primary importance for inducing SE (Fehér, 2015). During many types of SE, the inductive stimuli evoke endogenous auxin biosynthesis, leading to increased auxin levels and inducing totipotency in somatic cells (Braybrook and Harada, 2008). In Arabidopsis, BBM transcriptionally regulates *LEC1* (Horstman et al., 2017) and *YUC3* and *YUC8* (Li et al., 2022b) and LEC1 transcriptionally regulates *YUC10* (Junker et al., 2012). All these relationships act directly on endogenous IAA biosynthesis as the YUCs function as flavin-containing monooxygenases to catalyze indole-3-pyruvate acid (IPyA) to form IAA (Stepanova et al., 2011). Besides, enzymatic activity of tryptophan pyruvate aminotransferase (TAA1) converts tryptophan into the intermediate product indole-3-pyruvic acid (IPyA), which is also a key rate-limiting step of IAA biosynthesis. In this study, IAA was found to be highly accumulated in EC (**Supplementary Table 3**), which may be attributed to the higher expression level of one *YUC* gene (Lk07754) and one *TAA1* gene (Lk22753) in EC. IAA participation in maintenance of multiplying cells has been demonstrated in SE of rubber tree (*Hevea brasiliensis*), and the higher accumulation of IAA in EC compared to NEC have been reported in Douglas-fir (*Pseudotsuga menziesii*) and Tamarillo (*Solanum betaceum*) (Gautier et al., 2019; Caeiro et al., 2022). Additionally, it has been reported that the increase in endogenous auxin biosynthesis play a role in determining developmental cell fate and in activating the proliferative activity of proembryo cells (Pérez-Pérez et al., 2019). These results may indicate that the maintenance of embryogenic potentials of EC is largely dependent on the high production of endogenous IAA. Consistent with the higher content of IAA in EC, we found that L-tryptophan, the precursor of IAA, also showed a higher accumulation in EC (**Tables S3**). To sum up, all these results may suggest a mechanism in the transdifferentiation during EC induction and the maintenance of embryogenic potentials of EC in *L. kaempferi*: on 2,4- D treated culture medium, the expression of cell-totipotency-related TF genes, *BBM*, were induced, probably by ARFs. BBM furtherly activated the expression of *LEC1*. The two TFs can in turn promote IAA biosynthesis by inducing *YUCs* expression, thereby forming a feed-forward loop to reinforce cell-fate maintenance and transition (Wang et al., 2022a). Additionally, *WUS* genes maintained high expression in EC, which may play a critical role in inducing somatic embryo on PGRs free medium.

### Plant hormone signal transduction

In EC, the expression levels of many auxin-related genes were highly expressed in EC (**Figure 7**), indicating the important role of these auxin-responsive genes in *L. kaempferi* EC induction and embryogenic potential maintenance. We found that more auxin-related genes including *AUX1s*, *AUX/IAAs*, *ARFs*, *GH3s*, and *SAURs* showed higher expression levels in NEC than in EC, but the upregulation of these genes may be induced by the 2,4-D in the culture medium (Ji et al., 2015). In auxin-related responses, these TFs that control the target gene expression in response to auxin were indicated as playing a central role (Teale et al., 2006). In Arabidopsis, an expression analysis revealed that six *ARFs* were significantly upregulated, whereas five other *ARF* genes were downregulated in SE-induced explants (Wojcikowska and Gaj, 2017). Evaluation of SE efficiency from the ARF mutants further suggests that multiple ARFs redundantly contribute to SE in Arabidopsis (Wang et al., 2022a). Additionally, one possibility is that an unknown Aux/IAA-ARF complex binds to totipotency-related TF gene loci in somatic cells and the subsequent recruitment of the TOPLESS-histone deacetylase complex prevents totipotent gene expression (Wu et al., 2015). Therefore, the differential expression of totipotency-related genes and the differential accumulation of IAA between EC and NEC may be due to different *ARF* genes that are expressed in EC and NEC, which may be a possible reason leading to the differences in embryogenic potential between EC and NEC cells. However, the precise molecular mechanisms require further study.

Cytokinin plays key roles in the initiation and further development of embryogenic cultures (Cabrera-Ponce et al., 2018; Gautier et al., 2019). In this study, we observed that many genes related to zeatin biosynthesis and cytokinin signal transduction were differentially expressed between EC and NEC. In cucumber (*Cucumis sativus* L.), *tRNA isopentenyltransferase* genes (*IPT*) were found to be upregulated in EC compared to NEC and both IAA and CTK was promoted in EC, which led to the partial activation of related signal transduction pathway (Xue et al., 2022). However, the regulatory effects of genes involved in IAA and zeatin biosynthesis and signal transduction pathways on EC induction and maintenance are in a complex and comprehensive manner. Further research is needed in *L. kaempferi* SE.

It was of interest to note that it might be expected that, because 2,4- D and 6-BA were supplied in the medium, genes involved in auxin and cytokinin signal transduction pathways would be the only prominently featured hormone-related genes. However, this was not the case. We found that genes related to JA and ETH biosynthesis and signal transduction pathways were also differentially expressed between EC and NEC. Our results also showed that JA had a higher accumulation in NEC and many genes involved in JA biosynthesis and signal transduction were differentially expressed between EC and NEC, with more genes upregulated in NEC (**Figure 7**). For ETH related genes, most DEGs related to ETH biosynthesis and signal transduction showed higher expression levels in NEC (**Figure 7**). JA regulates diverse morphogenetic processes and defense responses in plants (Ruduś et al., 2009) and ETH is commonly involved in stress and development responses (Mantiri et al., 2008). Therefore, observed changes in JA and ETH content and the differential expression of related genes suggest that NEC may be under stress (Gautier et al., 2019).

### Carbohydrate and amino acid metabolism

In primary metabolic aspects, “starch and sucrose metabolism” and “biosynthesis of amino acids” were the representative pathways involving a lot of DEGs and DAMs (**Supplementary Table 12**, **13**). These results may indicate that both EC and NEC had vigorous primary metabolic activities. Starch and sucrose metabolism is closely related to cell division, tissue differentiation and organ formation (Eveland and Jackson, 2012; Xue et al., 2022) and may play a key role in determining embryogenesis (Baskaran et al., 2016). Previous studies have reported the differences in starch and sucrose content between EC and NEC. The higher accumulation of starch in cucumber EC may be the necessary prerequisite for further division of embryogenic cells (Xue et al., 2022). However, this seems not the case for Douglas-fir― starch content in NEC were significantly higher than that in EC, indicating that NEC was oriented toward energy storage in starch (Gautier et al., 2019). Although the metabolomic data provided limited information about saccharide metabolites, the large number of DEGs related to sugar metabolism may reflect the differences in carbohydrate and energy metabolism between EC and NEC. Additionally, many DEGs and DAMs between EC and NEC were related to the biosynthesis of amino acids (**Supplementary Table 12**, **13**), which may indicate that the embryogenic potential may be affected by amino acid metabolism. It has been suggested that the content of free proline in callus may affect tissue differentiation, and the callus with higher content of free proline has stronger capacity of SE (Gerdakaneh et al., 2011; Xue et al., 2022). Phenylalanine and tryptophan are precursors for secondary metabolites and IAA, respectively. The higher accumulation of these amino acids may be due to the requirement of EC cells for frequent cell division and differentiation (Ng et al., 2016).

### Molecular regulation of phenylpropanoid and flavonoid biosynthesis in *L. kaempferi* callus

In our results, flavonoids and phenolic acids accounted for 39% of all the DAMs (20.41% and 18.59%, respectively) (**Table 1**), and most of them were upregulated in NEC (**Supplementary Table 4**), which indicated that, compared to EC, more vigorous secondary metabolic activities may take place in NEC and thus led to the higher accumulation of flavonoids and phenolic acids in NEC. In contrast, primary metabolic activities were robust in both EC and NEC. In Douglas fir, 48 transcripts involved in the production of flavonoids were upregulated in NEC, and such increases in NEC were reflected by the tissue browning of NEC (Gautier et al., 2019). It was found that the secondary metabolites’ content, including anthocyanin, chlorophyll, total phenols, and flavonoids, induced in EC was lower than those in NEC in cotton embryogenesis (Zhang et al., 1992), and the higher accumulation of flavonoids in NEC was also detected in another report on cotton SE (Guo et al., 2019). It was also found that the active secondary metabolites may block the primary metabolism, resulting in delayed cell differentiation during SE (Sun et al., 2018). These findings suggest that the secondary metabolism levels in the NEC were higher, and the secondary metabolism affected cell division and differentiation by affecting the rate and intensity of the primary metabolism (Fan et al., 2022).

Flavonoids and phenolic acids are thought to contribute to the antioxidant potential in plants (Bajalan et al., 2016; Irakli et al., 2019). The biosynthesis of phenolic acids and flavonoids is conserved in plants, and the enzymes and genes involved in the two pathways have been well characterized in the last years (Zhou et al., 2019). Our results showed that most of the DEGs and DAMs were upregulated in NEC compared to EC, indicating that the biosynthesis of flavonoids and phenolic acids may play a critical role in *L. kaempferi* tissue transdifferentiation. The association analysis between metabolomics and transcriptomics is useful in identifying functional genes and elucidate the metabolic pathway of interest (Mao et al., 2021). The results of correlation analysis showed that the upregulation of multiple genes would ultimately result in the higher accumulation of several metabolites, which indicated that there is a complex regulatory mechanism between metabolite accumulation and gene expression in *L. kaempferi* calluses.

Many TFs have been proven to regulate phenylpropanoid or flavonoid biosynthesis in plants (Ma et al., 2018; Li et al., 2020a). For example, an ABA-responsive TF SmbZIP1 promotes phenolic acid biosynthesis by upregulation of expression of the biosynthetic gene *C4H1* in *Salvia miltiorrhiza* (Deng et al., 2020). It has been reported that R2R3-MYB, bHLH, and WD40 repeat protein play an essential role in regulating biosynthesis (Gu et al., 2019). In this study, we found that the higher expression of seven co-regulated genes including two *C4H* genes, two *CcoAOMT* genes, and three *HCT* genes (**Table 3**) involved in both phenylpropanoid and flavonoid biosynthesis pathways could result in the higher accumulation of phenolic acids and flavonoids in NEC. The targeted relationship between the predicted TFs and the key structural genes may affect the biosynthesis of phenolic acids and flavonoids. It was found that MYB15 in Arabidopsis is required for the activation of phenylpropanoid biosynthesis genes such as *PAL*, *C4H*, *4CL*, *HCT*, and *CAD* (Kim et al., 2020). It has also been reported that overexpression of *FTMYBs* in tartary buckwheat (*Fagopyrum tataricum*) resulted in higher accumulation of phenylpropanoid and anthocyanin accumulation in hairy roots and structural genes such as *PAL*, *4CL*, *C4H*, *CHI*, and *F3H* were markedly upregulated in transgenic lines (Li et al., 2020b). Additionally, the regulatory effects of ERFs on phenylpropanoid or flavonoid biosynthesis have been also addressed in many studies. For example, PhERF6 could interact with EOBI to negatively regulate fragrance (volatile benzenoids/phenylpropanoid) biosynthesis in petunia (*Petunia hybrida*) flowers (Liu et al., 2017). In mulberry (*Morus alba*), the expression level of *ERF5* gene showed high correlation with anthocyanin change pattern in the post-flowering stages and ERF5 could bind to the promotor regions of *F3H* and *MYBA* to transcriptionally activate their expression (Mo et al., 2022). The integrated transcriptome and metabolome analyses on two sugarcane (*Saccharum* spp.) varieties revealed that C2C2-dof may function as a hub gene in regulating phenylpropanoid biosynthesis, flavone and flavonol biosynthesis, and starch and sucrose metabolism (Yuan et al., 2022). These results supported our findings that there may be a potential targeting relationship between the four important TFs and several structural genes involved in phenolic acid and flavonoid biosynthesis, and their expression levels were significantly positively correlated with the target gene abundance (**Figure 10** and **11**). Therefore, one of our future tasks will focus on the validation of the functions of these TF genes and the target relationship between TFs and structural genes to furtherly reveal the relationship between second metabolite accumulation and embryogenic potential maintenance.

## Conclusion

Tremendous differences in transcriptomic and metabolomic profiling were observed between EC and NEC of *L. kaempferi*. The high-frequency cell division and differentiation of EC cells may be supported by the feed-forward loop composed of high expression levels of *ARF* genes, totipotency-related TF genes, IAA biosynthesis genes, and resulted high IAA production in EC. Carbohydrate metabolism, amino acid metabolism, and plant hormone synthesis and signal transduction may also play an important role in the maintenance of embryogenic potential. NEC featured upregulation of stress response, with more genes or metabolites involved in JA and ETH biosynthesis and transduction, and phenylpropanoid and flavonoid biosynthesis upregulated. By analyzing the matching relationship between the TFs and the promoter sequences of key structural genes and the correlations between their transcription levels, we found that several TFs including ERF, MYB and C2C2-dof may be crucial in regulating phenolic acid and flavonoid biosynthesis. This work represents the first report providing integrated insights into transcriptional and metabolic events involved in EC induction and maintenance in conifer SE.

## Data availability statement

The original contributions presented in the study are publicly available. This data can be found here: NCBI, PRJNA884809.

## Author contributions

JW performed the experiment, processed and analyzed the data, and wrote the first draft of the manuscript. LZ designed the experiment, prepared the plant material, analyzed the data and and carried out the manuscript revision. LQ and SZ supervised the work and carried out the manuscript revision. All authors contributed to the article and approved the submitted version.

## Funding

This research was funded by the Research Fund of Key Laboratory of Tree Breeding and Cultivation of National Forestry and Grassland Administration (ZDRIF202101), the National Natural Science Foundation of China (32171811 and 31600544), and the National Transgenic Major Program of China (2018ZX08020-003).

## Acknowledgments

We would like to thank all colleagues and friends who have contributed to this study.

## Conflict of interest

The authors declare that the research was conducted in the absence of any commercial or financial relationships that could be construed as a potential conflict of interest.

## Publisher’s note

All claims expressed in this article are solely those of the authors and do not necessarily represent those of their affiliated organizations, or those of the publisher, the editors and the reviewers. Any product that may be evaluated in this article, or claim that may be made by its manufacturer, is not guaranteed or endorsed by the publisher.
