# Supplementary figures and images for: Integrated transcriptomic and metabolic analyses provide insights into the maintenance of embryogenic potential and the biosynthesis of phenolic acids and flavonoids involving transcription factors in Larix kaempferi (Lamb.) Carr
Source: Front Plant Sci. 2022 Nov 17;13:1056930. doi: 10.3389/fpls.2022.1056930 (PMC9714495; doi:10.3389/fpls.2022.1056930)

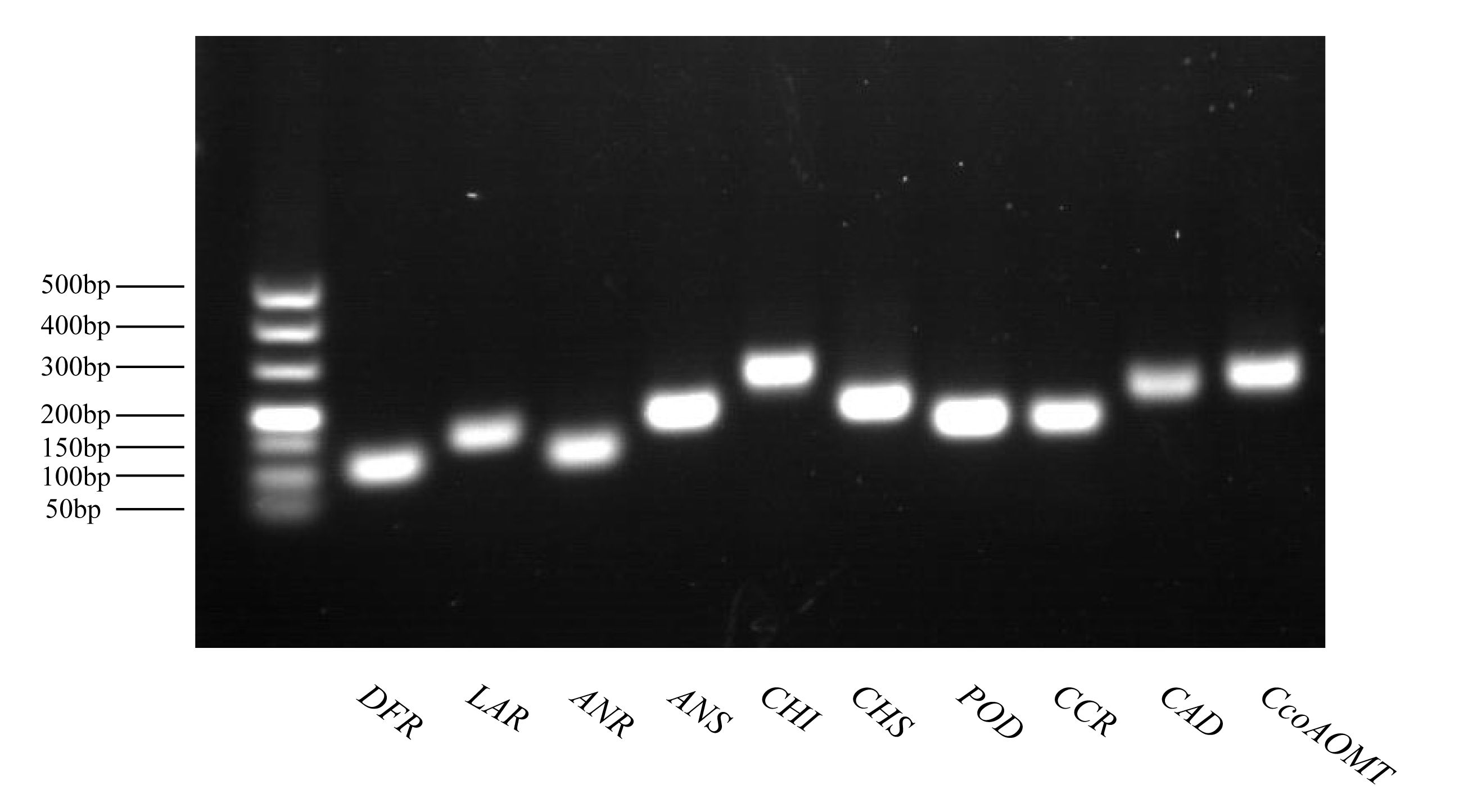

Supplement: Supplementary file 1 [file Image_1.tif]

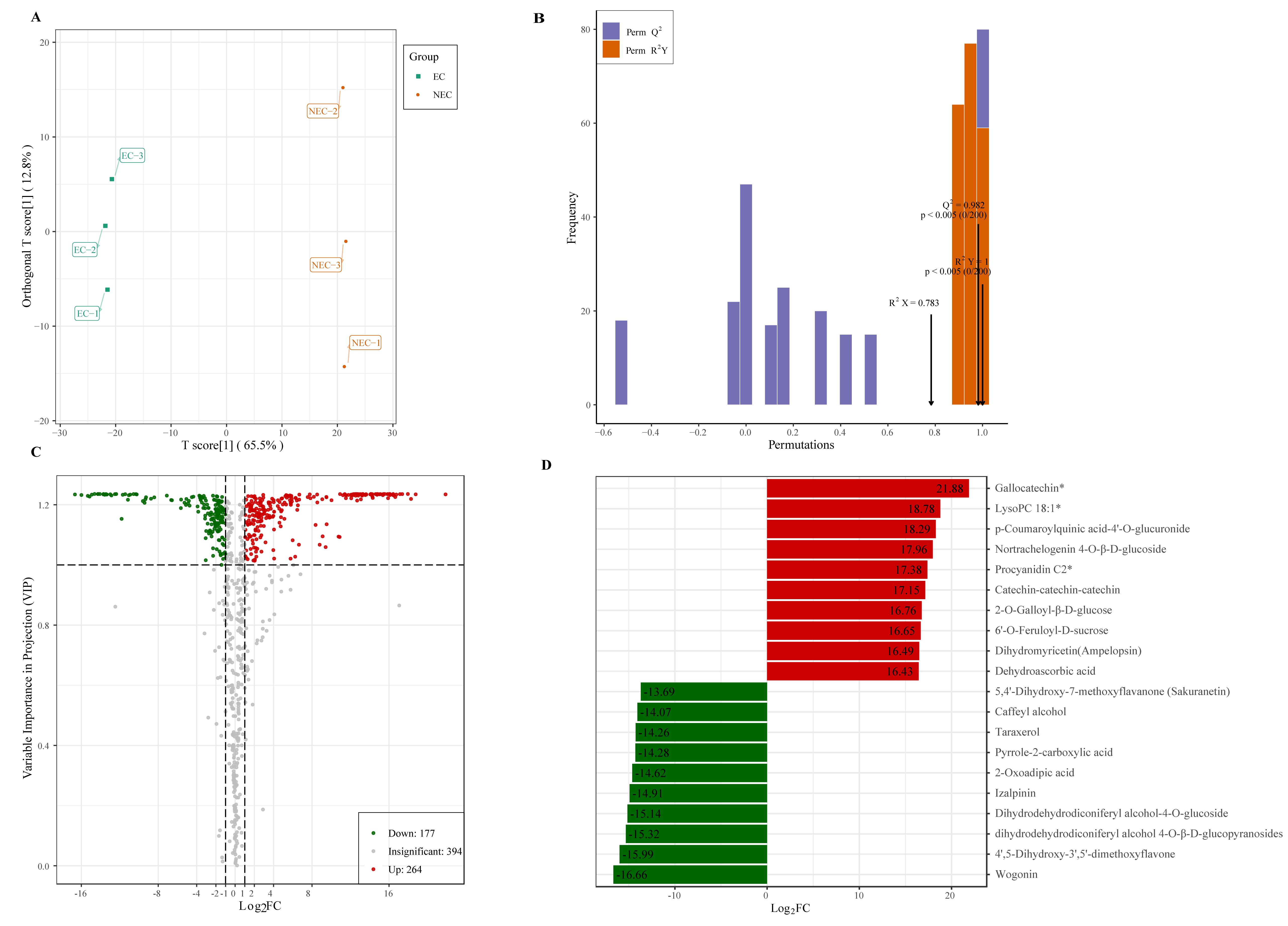

Supplement: Supplementary file 2 [file Image_2.tif]

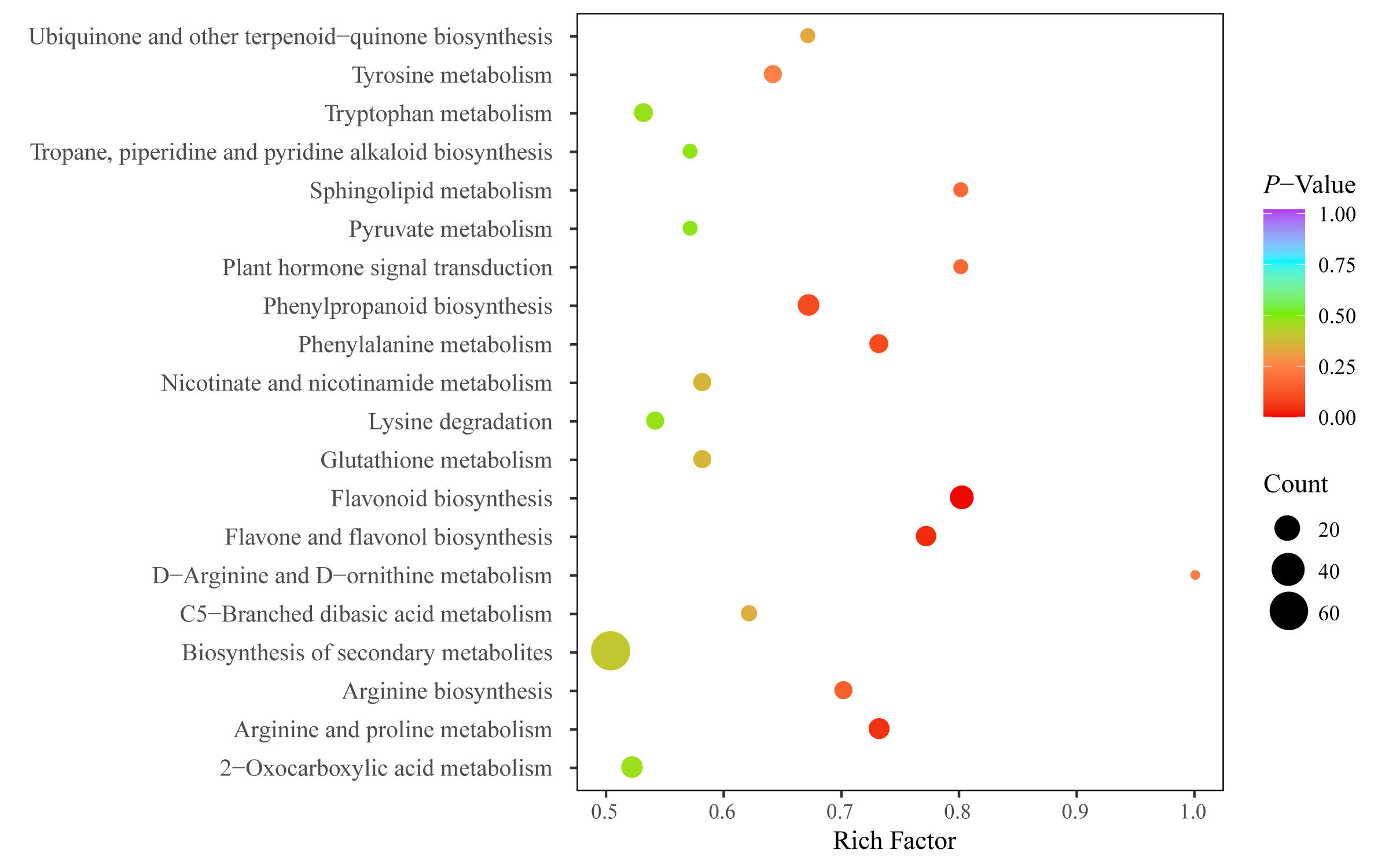

Supplement: Supplementary file 3 [file Image_3.tif]

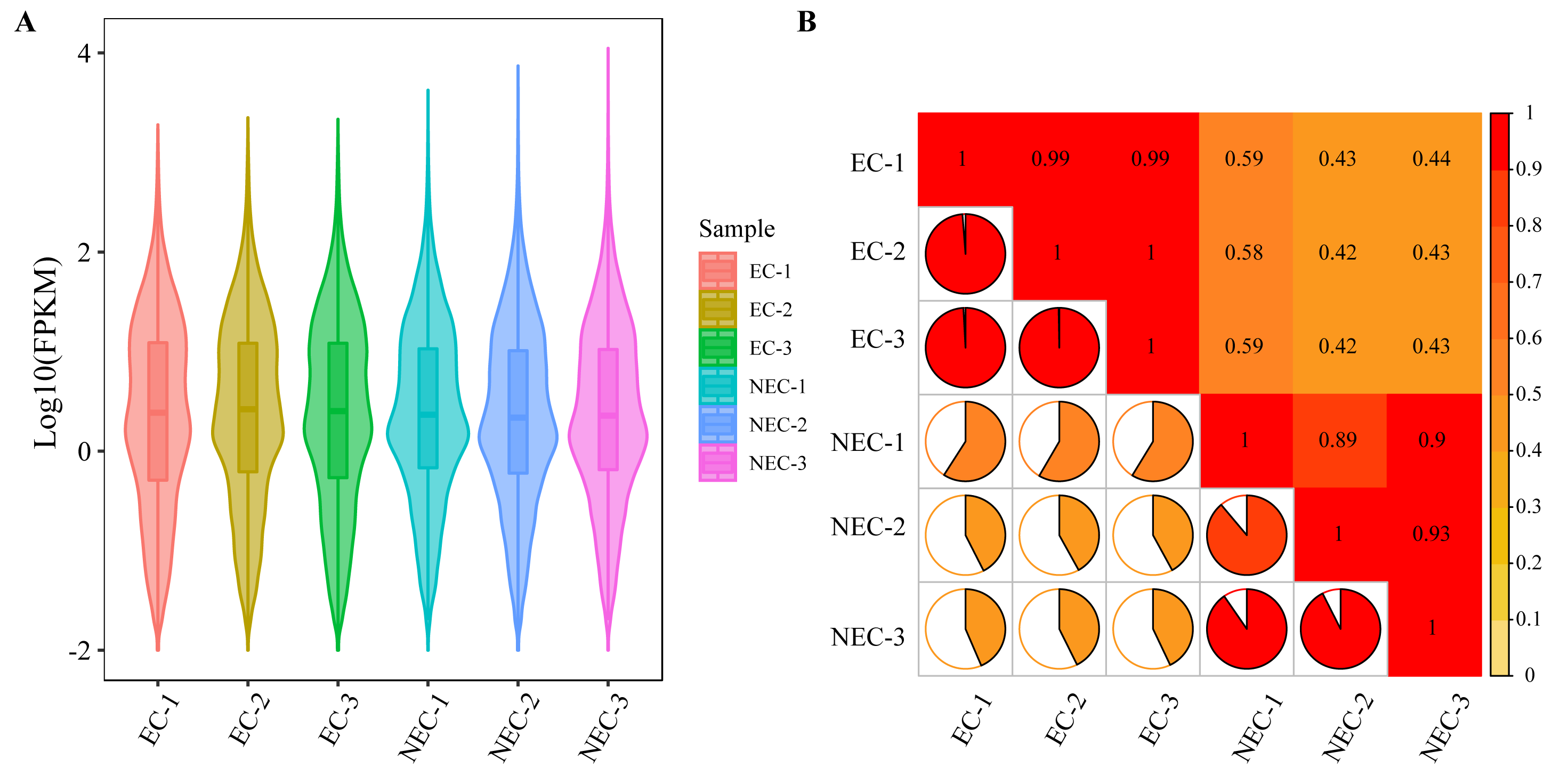

Supplement: Supplementary file 4 [file Image_4.tif]

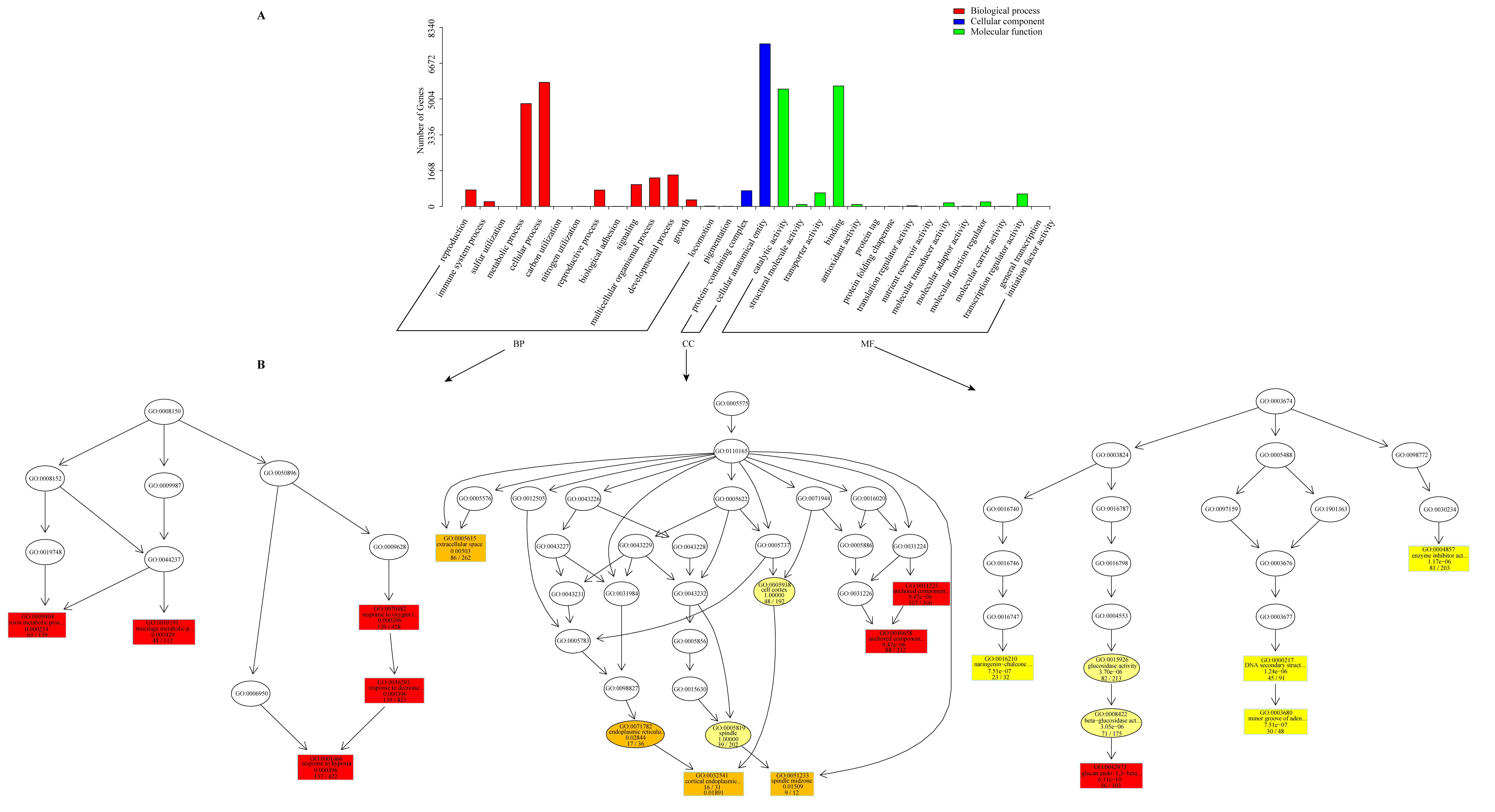

Supplement: Supplementary file 5 [file Image_5.tif]

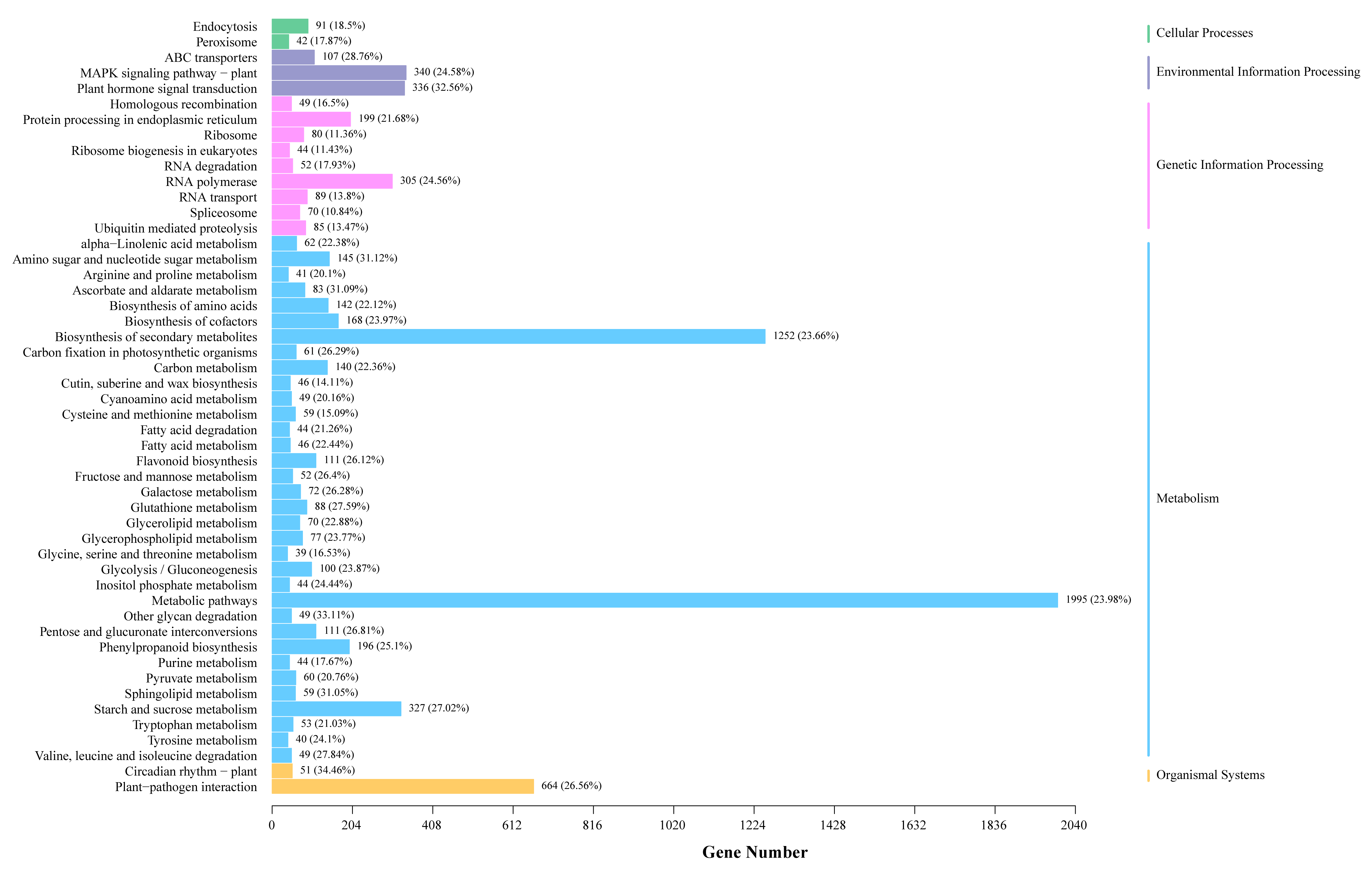

Supplement: Supplementary file 6 [file Image_6.tif]

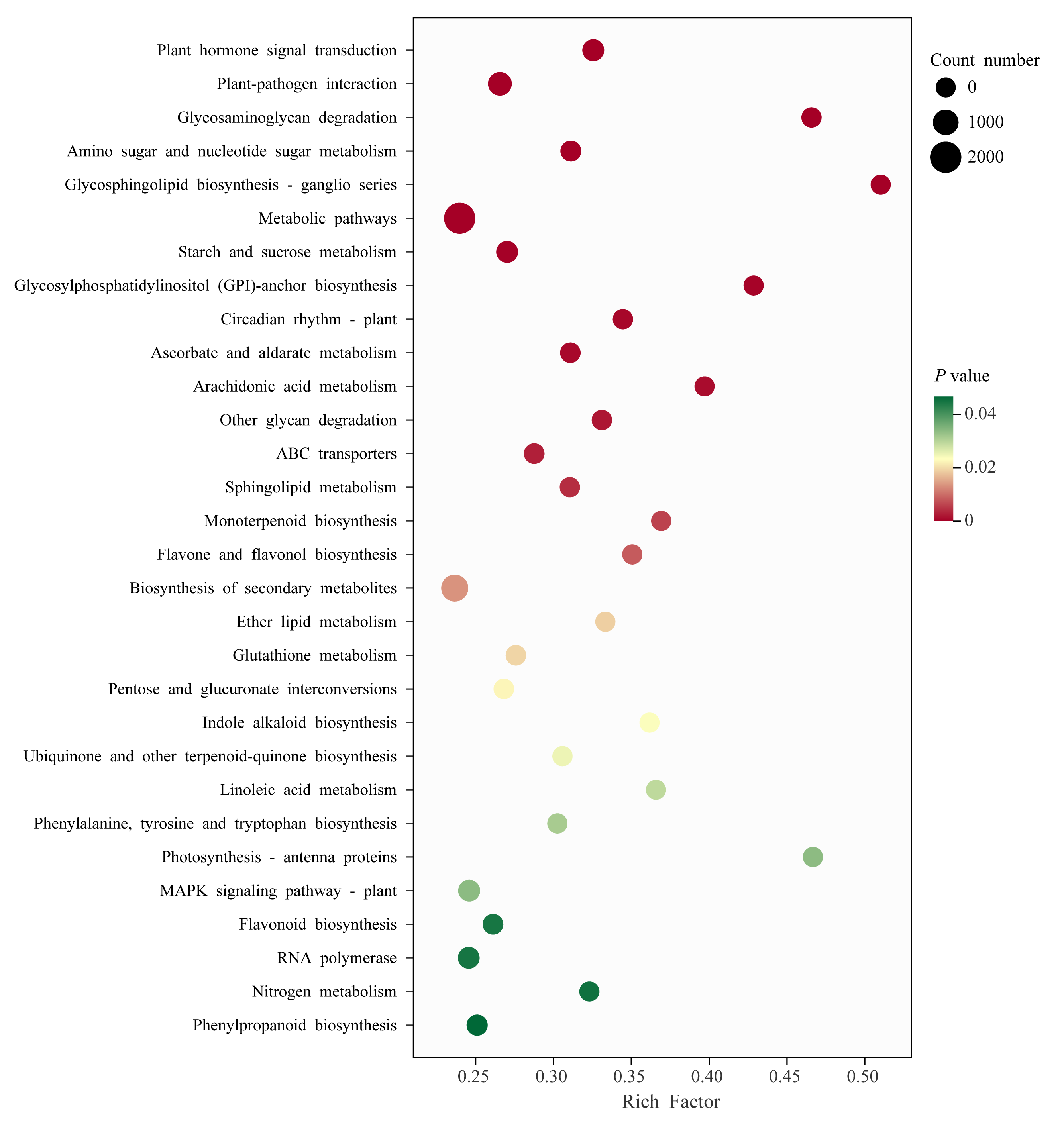

Supplement: Supplementary file 7 [file Image_7.tif]
