# Supplementary material for: Integrated transcriptomic and metabolic analyses provide insights into the maintenance of embryogenic potential and the biosynthesis of phenolic acids and flavonoids involving transcription factors in Larix kaempferi (Lamb.) Carr
Source: Front Plant Sci. 2022 Nov 17;13:1056930. doi: 10.3389/fpls.2022.1056930 (PMC9714495; doi:10.3389/fpls.2022.1056930)
Supplement: Supplementary file 8 [file Table_1.docx]

**Supplementary Table 1**. Categories of the 835 metabolites identiﬁed in EC and NEC of *L. kaempferi*

| Order | Category | Number of metabolites |
| --- | --- | --- |
| 1 | Lipids | 130 |
| 2 | Flavonoids | 128 |
| 3 | Phenolic acids | 123 |
| 4 | Amino acids and derivatives | 119 |
| 5 | Organic acids | 73 |
| 6 | Nucleotides and derivatives | 68 |
| 7 | Saccharides and Alcohols | 63 |
| 8 | Alkaloids | 45 |
| 9 | Lignans and Coumarins | 28 |
| 10 | Vitamin | 17 |
| 11 | Others | 16 |
| 12 | Terpenoids | 13 |
| 13 | Tannins | 11 |
| 14 | Quinones | 1 |
|  | Total | 835 |
