# Supplementary material for: Integrated transcriptomic and metabolic analyses provide insights into the maintenance of embryogenic potential and the biosynthesis of phenolic acids and flavonoids involving transcription factors in Larix kaempferi (Lamb.) Carr
Source: Front Plant Sci. 2022 Nov 17;13:1056930. doi: 10.3389/fpls.2022.1056930 (PMC9714495; doi:10.3389/fpls.2022.1056930)
Supplement: Supplementary file 9 [file Table_2.docx]

**Supplementary Table 2**. The primers used for qRT-PCR analysis

| **Gene ID** | **Symbol** | **Forward primer (5'-3')** | **Reverse primer (5'-3')** |
| --- | --- | --- | --- |
| Lk17760 | *CcoAOMT* | GGCTGCCTATCATTGAGAAAG | CCTCCAACCTTCACCAGAT |
| Lk36760 | *CAD* | TCAAGAATCAACACCCAGG | ACATAATGAGAGGGAGGCTATC |
| Lk31851 | *CCR* | GAAGCAACAACCCATTGG | GGAGCACTCTGAACTCGTAAC |
| Lk22889 | *POD* | TGTCAATCTCCTCAACAGGC | GAAAGAATAGGCTCTGGTTCAC |
| Lk04715 | *CHS* | AGCCCAAGAAACTGAGAGC | GAACTCCCACATCCAATCC |
| Lk45246 | *CHI* | AGGAAGAGGAAGCACTGGA | ACTGGGAGACAGCAGAGGAT |
| Lk00086 | *ANS* | GTGGAGGAAGAGAGAATGGA | CAGAAGGAATGTCAGTGCG |
| Lk13190 | *ANR* | TACAATGTCCCAACCCAGT | TTGAAGGAGAAACCGCTC |
| Lk22915 | *LAR* | GCCCTACTTATGCCCTTGT | TCAACCTGCCTTATGGCT |
| Lk11503 | *DFR* | GGACACACCACTACCATTCC | AGCAGCAACAGACTCATCG |
| Reference gene | *LkEF1A* | GACTGTACCTGTTGGTCGTG | CCTCCAGCAGAGCTTCAT |
